# Verbal Learning and Memory in Cannabis and Alcohol Users: An Event-Related Potential Investigation

**DOI:** 10.3389/fpsyg.2017.02129

**Published:** 2017-12-08

**Authors:** Janette L. Smith, Frances M. De Blasio, Jaimi M. Iredale, Allison J. Matthews, Raimondo Bruno, Michelle Dwyer, Tessa Batt, Allison M. Fox, Nadia Solowij, Richard P. Mattick

**Affiliations:** ^1^National Drug and Alcohol Research Centre, University of New South Wales, Sydney, NSW, Australia; ^2^School of Psychology, University of New South Wales, Sydney, NSW, Australia; ^3^School of Psychology and Illawarra Health and Medical Research Institute, University of Wollongong, Wollongong, NSW, Australia; ^4^School of Medicine, University of Tasmania, Hobart, TAS, Australia; ^5^School of Psychological Science, University of Western Australia, Perth, WA, Australia

**Keywords:** RAVLT, principal components analysis, recollection, familiarity, alcohol, cannabis

## Abstract

**Aims:** Long-term heavy use of cannabis and alcohol are known to be associated with memory impairments. In this study, we used event-related potentials to examine verbal learning and memory processing in a commonly used behavioral task.

**Method:** We conducted two studies: first, a small pilot study of adolescent males, comprising 13 Drug-Naive Controls (DNC), 12 heavy drinkers (HD) and 8 cannabis users (CU). Second, a larger study of young adults, comprising 45 DNC (20 female), 39 HD (16 female), and 20 CU (9 female). In both studies, participants completed a modified verbal learning task (the Rey Auditory Verbal Learning Test, RAVLT) while brain electrical activity was recorded. ERPs were calculated for words which were subsequently remembered vs. those which were not remembered, and for presentations of learnt words, previously seen words, and new words in a subsequent recognition test. Pre-planned principal components analyses (PCA) were used to quantify the ERP components in these recall and recognition phases separately for each study.

**Results:** Memory performance overall was slightly lower than published norms using the standardized RAVLT delivery, but was generally similar and showed the expected changes over trials. Few differences in performance were observed between groups; a notable exception was markedly poorer delayed recall in HD relative to DNC (Study 2). PCA identified components expected from prior research using other memory tasks. At encoding, there were no between-group differences in the usual P2 recall effect (larger for recalled than not-recalled words). However, alcohol-related differences were observed in a larger P540 (indexing recollection) in HD than DNC, and cannabis-related differences were observed in a smaller N340 (indexing familiarity) and a lack of previously seen > new words effect for P540 in Study 2.

**Conclusions:** This study is the first examination of ERPs in the RAVLT in healthy control participants, as well as substance-using individuals, and represents an important advance in methodology. The results indicate alterations in recognition memory processing, which even if not manifesting in overt behavioral impairment, underline the potential for brain dysfunction with early exposure to alcohol and cannabis.

## Introduction

Acute as well as chronic use of both alcohol and cannabis can result in memory dysfunction (see, for example, Solowij and Battisti, 2008; Konrad et al., 2012; Crane et al., 2013; Schoeler and Bhattacharyya, 2013; Bernardin et al., 2014; Broyd et al., 2016). Recent research has focused on the possible effects of younger age of onset of use (e.g., Pope et al., 2003; Wagner et al., 2010; Crane et al., 2015), dose-dependent effects in recreational vs. heavy users (e.g., Chye et al., 2017), and the possibility of recovery with abstinence (e.g., Yücel et al., 2016).

In this study we focus on a well-known test of verbal learning and memory, the Rey Auditory Verbal Learning Test (RAVLT; Rey, 1941; Lezak et al., 2004). The RAVLT tests memory for 15-item lists of unrelated words and allows for measurement of learning across five trials (Trials I-V), followed by recall of a second list (Trial B), and then immediate (Trial VI) and delayed recall (Trial VII), and recognition of the initial list. The RAVLT is widely used, easy to administer, and has published norms available (e.g., Vakil et al., 1998, 2010; Carstairs et al., 2012).

Regular cannabis users have been shown to perform more poorly than non-using controls on the RAVLT and related memory tasks when not acutely intoxicated (for review see Broyd et al., 2016). Impairments have been reported by our team for both adult (Solowij et al., 2002) and adolescent cannabis users (Solowij et al., 2011). Cannabis-related deficits in memory and learning appear not to be permanent (e.g., Pope et al., 2001; Broyd et al., 2016), with meta-analytic reviews suggesting that only small magnitude effects are apparent in the first few weeks of abstinence (of the order of d = 0.25 to 0.35), and these become smaller and non-significant with extended abstinence (to around d = 0.15; Schreiner and Dunn, 2012).

There are disparities in the reported results for alcohol dependent groups or heavy drinkers in comparison to controls. For alcohol dependence, Phelan (2013) reported fewer words recalled over Trials I-V for alcohol dependent participants (approaching significance), and alcohol dependence was also associated with poorer recognition performance. On the other hand, Waugh et al. (1989) report intact performance over Trials I-V, but significantly poorer performance for heavy drinkers consuming 81–130 g of alcohol per day on Trial V and VI. A meta-analytic study of alcohol dependence reports deficits with medium effect sizes that do not fully recover with extended abstinence (>365 days; Stavro et al., 2012). Amongst young heavy drinkers, Parada et al. (2011) report greater proactive interference, while Winward et al. (2014) reported impairments in delayed recall despite similar initial memory performance. However, our team has found no differences between adolescent drinkers and non-drinkers in RAVLT performance (Solowij et al., 2011), while Kokavec and Crowe (1999) reported trends toward *better* performance among binge drinkers.

In the current study, we examine in detail the memory performance of groups of young heavy drinkers, cannabis users (most, but not all, of whom were also heavy drinkers), and controls who neither used cannabis nor drank heavily. In addition to studying behavioral measures, we also examine electrophysiological functioning using event-related potentials or ERPs. These represent the brain's average electrical response to an event, resulting in peaks and troughs of electrical negativity and positivity corresponding to various stages of processing, reviewed below. In several studies, electrophysiological and neuroimaging measures have proven to be more sensitive to drug effects than behavioral measures (e.g., Solowij et al., 1995; Maurage et al., 2009; Norman et al., 2011; Mahmood et al., 2013) and may indicate subtle deficits in processing which are not yet strong enough to influence gross measures such as error rates and reaction time. Our group has previously reported differences in ERPs associated with word list learning between light and heavy drinkers in the absence of behavioral performance differences (Fox et al., 1995), and also ERP alterations and verbal memory deficits in chronic cannabis users (Battisti et al., 2010).

Despite the importance of studying brain function to identify subtle or underlying processes, only two papers have examined ERPs in the RAVLT (Babiloni et al., 2009, 2010). Babiloni et al. (2009) recorded intracerebral electrical activity in patients with temporal lobe epilepsy during the recall phase of the RAVLT, and examined event-related synchronization in the theta band for words which were recalled vs. words which were not recalled. In 2010, they presented traditional ERP analyses of the same participants, with a late positive peak apparent around 350 ms post-stimulus being larger for recalled than unrecalled words. While these results are in line with expectations for memory tasks as reviewed below, they are not easily generalizable to a wider population. Firstly, as epilepsy patients have abnormal patterns of brain activity, it is difficult to predict the pattern of brain activity in healthy control participants, much less potential differences in substance abusing individuals. Secondly, intracerebral recording techniques are less sensitive to noise than scalp-recorded ERPs. Lastly, presumably because of time and posture constraints associated with neurosurgery, the recognition portion of the RAVLT was not performed.

It is likely that the RAVLT has not been used in other ERP studies due to signal:noise ratio (SNR) difficulties. SNR is a function of both the size of the signal and the number of trials available for averaging, and as a rule of thumb, the largest ERP components may require 30–60 trials per condition to achieve adequate SNR, while the smallest (e.g., brainstem auditory evoked potentials) may require several thousand (Luck, 2004). Thus, the RAVLT has too few trials to produce reliable ERPs with acceptable SNRs for analysis via traditional methods.

The current study, however, uses established statistical procedures which can identify latent sources of variability in ERP waveforms. In general, principal components analysis (PCA) is a technique used to extract latent variables explaining variance in a dataset. When applied to ERPs in the temporal domain, PCA extracts factors which explain a large proportion of variance across time between subjects, conditions, and scalp sites, while noise, explaining a smaller proportion of variance, is reduced (Donchin and Heffley, 1978; Coles et al., 1986). Factor loadings can be analyzed to determine the time over which a particular component is active, while the peak component amplitudes for each identified factor of interest (analogous to the more traditional peak-picked component amplitudes) can be assessed statistically (via ANOVA or MANOVA) to examine potential differences in scalp distribution, and between conditions and groups.

One major difference in ERP waveforms associated with recall is the amplitude of the P2 component, being larger to words which are later recalled, compared to those which are not recalled (e.g., Chapman et al., 1978; Smith, 1993; Babiloni et al., 2010). Peak or mean amplitude measures have often been employed, despite Chapman et al. (1978) noting that the P2 related to memory overlaps in time with an earlier positive peak of the evoked potential, and that PCA-derived rather than peak measures of the grand average ERP may capture the P2 recall effect more precisely. We expect to observe similar differences in PCA-derived ERP measures in the recall phase of the RAVLT, for words which are vs. are not recalled.

Secondly, we expect to observe in the recognition phase of the RAVLT two major effects known as the parietal old/new effect and the frontal familiarity effect. Early studies on recognition memory reported more positive-going waveforms for previously studied (old) words compared to new words (e.g., Sanquist et al., 1980; Warren, 1980). However, later studies reported dissociation between effects at frontal vs. parietal sites, supporting a dual-process model of recognition memory, which asserts that recognition judgements may be made based on two types of information: familiarity (remembering) and recollection (knowing). Familiarity-based recognition involves a global matching process between study items and test items, while recollection requires a distinct memory signal involving the retrieval of the context of learning (for a review see Wilding and Rugg, 1996; Curran, 2000; Rugg and Curran, 2007). The ERP index of recollection is the parietal old/new effect, a parietally maximal positivity occurring 400–800 ms post-stimulus (here termed P600), often larger in the left hemisphere, and larger for previously studied words compared to new words. The index of familiarity is held to be the N400, a negativity occurring around 300–500 ms post-stimulus, typically at mid-frontal sites, which is more negative (larger) for new words. These effects have been functionally separated by experimental paradigms more complex than the RAVLT (e.g., Rugg et al., 1998; Curran, 2000), but based on those results we can predict different familiarity and recollection effects in the recognition phase of the RAVLT for List A, List B and New words (see Method for details).

The current studies build upon previous work examining memory in young heavy drinkers and cannabis users by including the first analysis of ERPs in addition to behavioral performance during the RAVLT. In a small pilot study of male adolescents, recorded with a reduced scalp montage, we first show proof of concept, that even with low numbers of trials, we can extract meaningful components from the ERPs which behave in predictable ways. In a subsequent larger study of young adults of both sexes, with a larger scalp montage, and more detailed information about use of alcohol, cannabis, and other drugs, we again demonstrate the viability of examining ERPs in the RAVLT (and that ERPs may be more sensitive to effects of alcohol and cannabis use, and of sex, than behavioral measures alone). To foreshadow the results, consideration of ERPs adds sensitivity to the analyses, since some substance-related differences were observed in ERP comparisons which were not apparent in behavioral data.

## Study 1

### Methods

#### Participants

Participants were 33 males (aged between 16 years and 18 years 11 months) recruited from a larger, separate cohort of adolescents (Mattick et al., 2017) who since age 12 have reported yearly on their use of alcohol and other substances. Participants were eligible to participate if they were not regular users of any other drug apart from alcohol, cannabis or tobacco, had normal or corrected vision, were not using psychoactive medications, and had never suffered a seizure or serious head injury. We recruited participants with a range of alcohol and cannabis consumption patterns, although because the sample sizes are small, and use of alcohol and/or cannabis was relatively low, we report exploratory analyses of drug-related effects in Supplementary Material only. All participants gave written informed consent, and the protocol was approved by the University of New South Wales Human Research Ethics Committee before data collection began in an EEG laboratory at the University of Tasmania.

### Procedures

The experimenter showed the participant the lab and recording equipment and described the experimental protocol before written informed consent was obtained. Participants then completed a short demographics questionnaire as well as questions about their alcohol and other drug use, and the Wechsler Test of Adult Reading (Holdnack, 2001).

A modified version of the RAVLT (Rey, 1941) was administered with standard instructions and word lists (i.e., drum, curtain, bell etc. for List A, and desk, ranger, bird, etc. for List B; Lezak et al., 2004). Because we wanted to use the words traditionally included in the RAVLT, but also to standardize the duration of their presentation, in line with many other ERP memory studies, we used a visual rather than auditory presentation modality. Participants were presented with the 15 List A words, displayed for 200 ms, with a 1000 ms stimulus onset asynchrony, in white capital letters on a black screen. Two seconds after the end of each sequence of 15 words, the word RECALL appeared in green text, prompting participants to recall, out loud, as many words as possible in any order. This was repeated five times (Trials I-V). Next, the 15 List B words were presented, with the same timing and instructions (Trial B). Following this, participants were unexpectedly asked to recall as many List A words as possible, without another presentation of that list (Trial VI). Participants then completed a 20 min non-verbal distractor task, followed by again being unexpectedly asked to recall List A words (Trial VII).

For the recognition part of the experiment, some further modifications were necessary for compatibility with ERP techniques. The usual method for the recognition phase is to present the 15 List A words, 15 List B words, and 20 new words in random order on a sheet of paper, and ask the participant to circle the List A words. Here, we presented the words one at a time, in white capitals on a black background, and asked participants to press one button (e.g., with the left hand) for List A words, and a different button (e.g., with the right hand) for “Other” words (i.e., List B and New words). The response assignment was counterbalanced between participants. Words were displayed on the screen until the participant made a response, and were then replaced by a black screen for 500 ms, when the next word appeared. For recall performance, we counted the number of words correctly recalled on Trials I-VII; we gave credit for words that were pluralized. For the recognition phase, we counted the number of words correctly categorized as List A/Other, and the time taken to make the response, for List A, List B and New words.

### EEG recording and analysis

Continuous monopolar EEG was recorded from 30 scalp sites using an elasticised cap with sintered Ag/AgCl electrodes. Additional electrodes recorded vertical and horizontal EOG. All electrodes were referenced to linked mastoids and grounded midway between FPz and Fz. Electrode impedances were below 5 kΩ. Signals were recorded between 0.05 and 30 Hz, and sampled at 1,000 Hz using NeuroScan recording software and hardware.

The EEG was filtered with a bandpass from 0.1 Hz (down 12 dB/octave) to 24 Hz (down 24 dB/octave, zero phase shift), and then corrected for eye movements using NeuroScan's inbuilt procedure (Semlitsch et al., 1986). Noisy electrodes were interpolated offline using Curry 7; 6 participants had one interpolated channel, 4 participants had two, and 1 participant had three. All epochs began 100 ms prior to and ended 900 ms after stimulus presentation, and were baselined during the prestimulus interval. Epochs were rejected if amplitude exceeded ±100 μV in any scalp channel. For ERPs in the recall phase, we created average ERPs for the presentation of words in Trials I-B which were “Remembered” or “Not Remembered” in the immediately subsequent recall period (Babiloni et al., 2010). An average of 47 trials (minimum 30) were included for Remembered words, while an average of 39 trials (minimum 21) were included for Not Remembered words. For the recognition phase, we created average ERPs to correctly categorized “List A,” “List B,” and “New” words. One participant performed poorly on the recognition task such that only 3 trials were available for averaging for List A words; this participant was excluded from analyses of ERPs from the recognition phase. For the remaining participants, an average of 13 List A trials (minimum 10), 14 List B trials (minimum 9), and 18 New trials (minimum 13) were included in the ERPs, representing more than 97% of the available trials for all three trial types.

### Data reduction

ERP data were downsampled to 200 Hz to increase the ratio of cases (subjects, conditions, sites) to variables (timepoints) and were then subjected to separate temporal principal components analyses (PCA) for the recall and recognition phases of the experiment, using Matlab 9.2 (R2017a) and the ERP PCA Toolkit (v2.53; Dien, 2010). Each PCA used the covariance matrix, Kaiser normalization, and varimax rotation (Kayser and Tenke, 2003, 2006; Dien et al., 2005), and Horn's Parallel Test (Horn, 1965) was used to identify the number of factors to be extracted and rotated. The Recall PCA (Remembered and Not Remembered trials) had 1980 cases (33 participants × 2 conditions × 30 sites), and was restricted to 14 factors which together accounted for 92.51% of variance. Factors were labeled based on their polarity and peak latency. Three positive factors were identified in the P2 time range, however only Factor 6, labeled P175 (peaking at 175 ms, maximal at FCz, and explaining 4.4% of unique variance) showed the expected Remembered > Not Remembered effect and thus is the only P2 component discussed here. For completeness, the additional factors peaking in the P2 range, as well as factors peaking at 440 ms (N400 time range) and 630 ms (P600 time range) are presented in Supplementary Material.

The Recognition PCA (on List A, List B and New words) had 2880 cases (32 subjects × 3 conditions × 30 sites), and was restricted to 13 factors which together explained 93.72% of variance. Factor 1, labeled P640 (peaking at 640 ms, maximal at CPz, explaining 24.76% of variance) was identified as reflecting the classical parietal Old/New (P600) effect, while Factor 2, labeled N415 (peaking at 415 ms, maximal at C4, explaining 18.41% of variance) was identified as reflecting the frontocentral N400 effect.

### Statistical analysis

For recall performance, several separate within-subject MANOVAs were performed. To assess differences in learning rate over Trials I-V, we ran a MANOVA with Trial as a factor (I, II, III, IV, V); polynomial contrasts on the trial factor assessed the change over trials, although only linear and quadratic trends were examined. To assess proactive interference (i.e., poor recall of new material due to interference from learning of old materials), we compared Trial I with Trial B. For assessment of retroactive interference (i.e., poor recall of old material due to interference from learning of new material), we compared Trial V with Trial VI. We assessed forgetting over time by comparing Trial V with Trial VII. Descriptive statistics only (means and standard errors) were calculated for the accuracy and reaction time for correctly categorized words in the Recognition phase.

Peak component amplitudes from the sites F3, Fz, F4, C3, Cz, C4, P3, Pz, and P4 were each assessed with three-way MANOVAs with factors Lateral (left/midline/right), Sagittal (frontal/central/parietal) and Type (for the recall phase: Remembered, Not Remembered; for the recognition phase: List A, List B, New). Contrasts on the Sagittal factor compared activity at frontal sites with that at parietal sites, and their average with activity at central sites. Contrasts on the Lateral factor compared activity at left hemisphere sites with that at right hemisphere sites, and their average with activity at midline sites. Such contrasts are optimal for efficiently deriving maximal information about component topography. For the recognition phase, planned contrasts on the Type factor for N415 (indexing familiarity) compared activity for List A vs. List B words (highly familiar words vs. less so), and their mean (words which had been presented before) vs. New (not seen before). For the P640 (indexing recognition), we compared List A words with the mean of List B and New words (indicating correct source recollection of the word as being List A vs. Other), and compared List B with New words (although this is necessarily confounded with familiarity). These analyses are important for characterizing the topographic distribution of the component, and differences in amplitude and topography between different trial types.

As the contrasts were planned and there were no more of them than the degrees of freedom for effect, no Bonferroni-type adjustment to alpha was necessary (Tabachnick and Fidell, 2001). Because this is a first step in examining ERPs in the RAVLT, with a small sample size and low power, but with an aim to report potential discoveries to spur future research, we report any effect with *p* < 0.100.

## Results and discussion

### Demographics

Participants' mean age was 17.2 years (SD = 0.7 years), and standardized scores on the WTAR were in the normal range (mean 102.7, SD = 16.5). Five participants were left-handed.

### Behavioral performance

**Figure 1:**
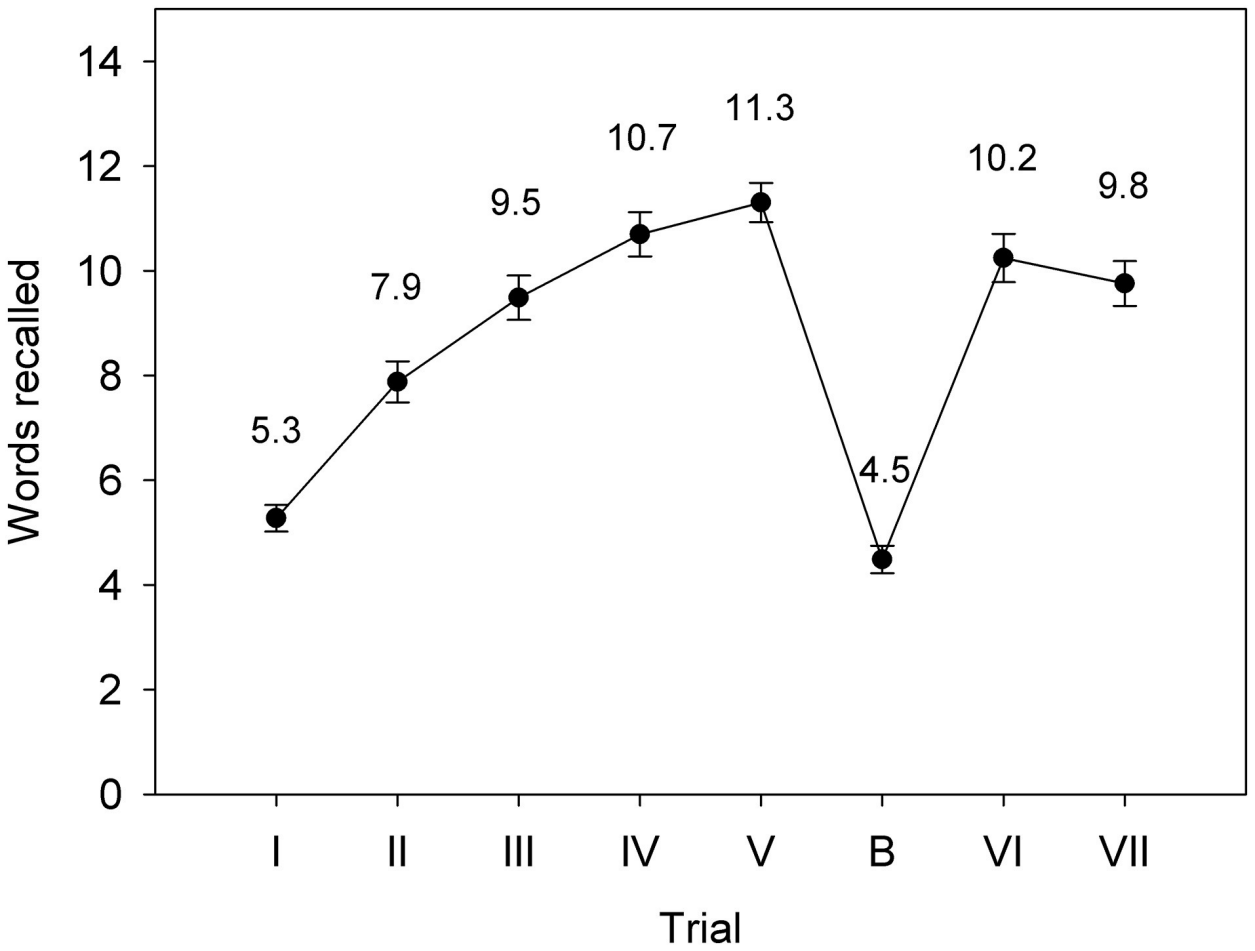
Mean RAVLT performance in the Recall phase for the adolescent males (Study 1). Error bars represent the standard error of the mean.

Figure 1 shows the mean number of words recalled by participants for each trial. There were highly significant increases in the number of words remembered over Trials I-V (linear trend *F* = 312.74, *p* < 0.001; quadratic trend *F* = 17.64, *p* < 0.001; for all effects reported in this section *df* = 1.32), indicating learning of words over trials. On average, participants remembered fewer words for Trial B than for Trial I (*F* = 4.77, *p* = 0.036), indicating proactive interference. Participants remembered significantly fewer words for Trial VI compared to Trial V (*F* = 9.91, *p* = 0.004), indicating retroactive interference. Participants remembered significantly fewer words for Trial VII than for Trial V (*F* = 20.64, *p* < 0.001), indicating forgetting after a delay of 20 min. Categorisation of List A, List B and New words was generally accurate (List A: mean = 13.5, SD = 2.3; List B: mean = 14.0, SD = 1.3; New: mean = 18.3, SD = 1.9). RT for correct categorisations was similar across trial types (List A: mean = 885.6 ms, SD = 309.4 ms; List B: mean = 879.9 ms, SD = 205.0 ms; New: mean = 932.4 ms, SD = 231.5 ms).

Despite our modifications to the RAVLT required for recording and analyzing ERPs, we observed the within-subject effects typically seen in the standard version—that is, learning over trials, proactive and retroactive interference, and forgetting after a delay. The slightly poorer performance of our group relative to published norms (e.g., Carstairs et al., 2012) may be a consequence of our decision to use the visual rather than auditory modality for stimulus presentation, since free recall is typically better for words presented verbally than in print (e.g., Murdock and Walker, 1969), at least for Trial I (van der Elst et al., 2005), as well as the lack of opportunity to revisit words in the recognition phase.

### Recall ERPs

**Figure 2:**
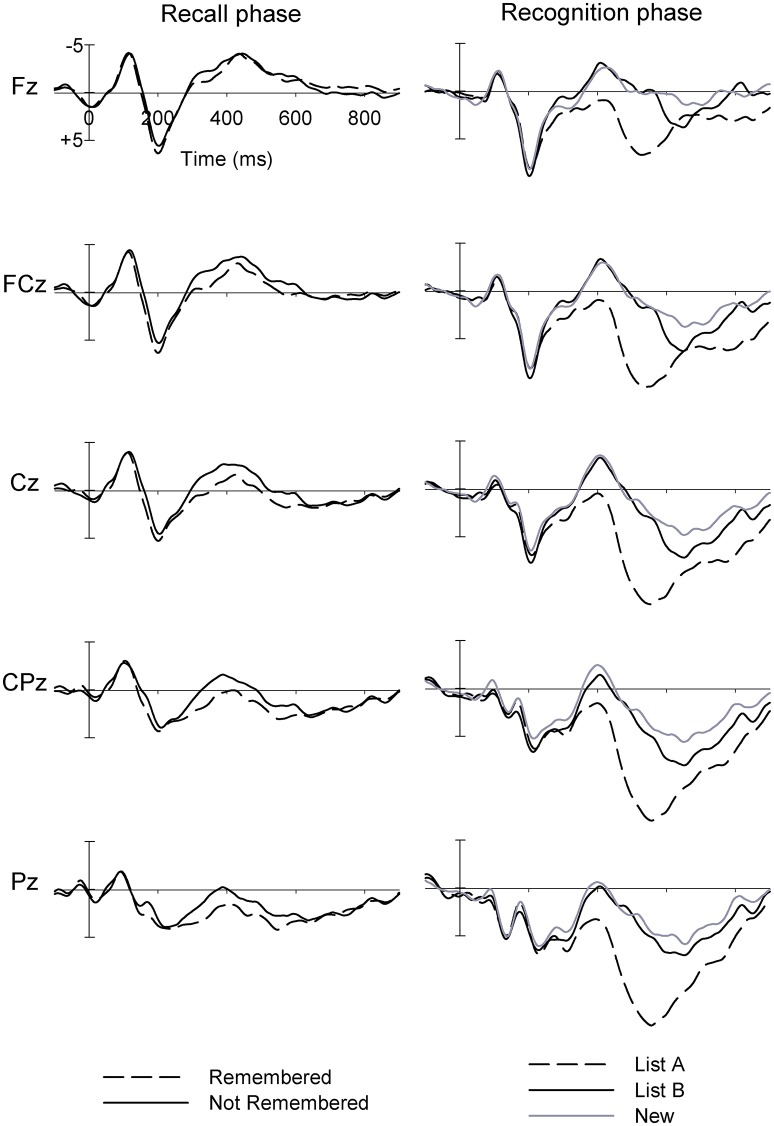
Grand mean ERPs at midline sites for (left panel) words which were later Remembered and Not Remembered in the Recall phase, and (right panel) List A, List B and New words in the Recognition phase, for adolescent males (Study 1).

**Figure 3:**
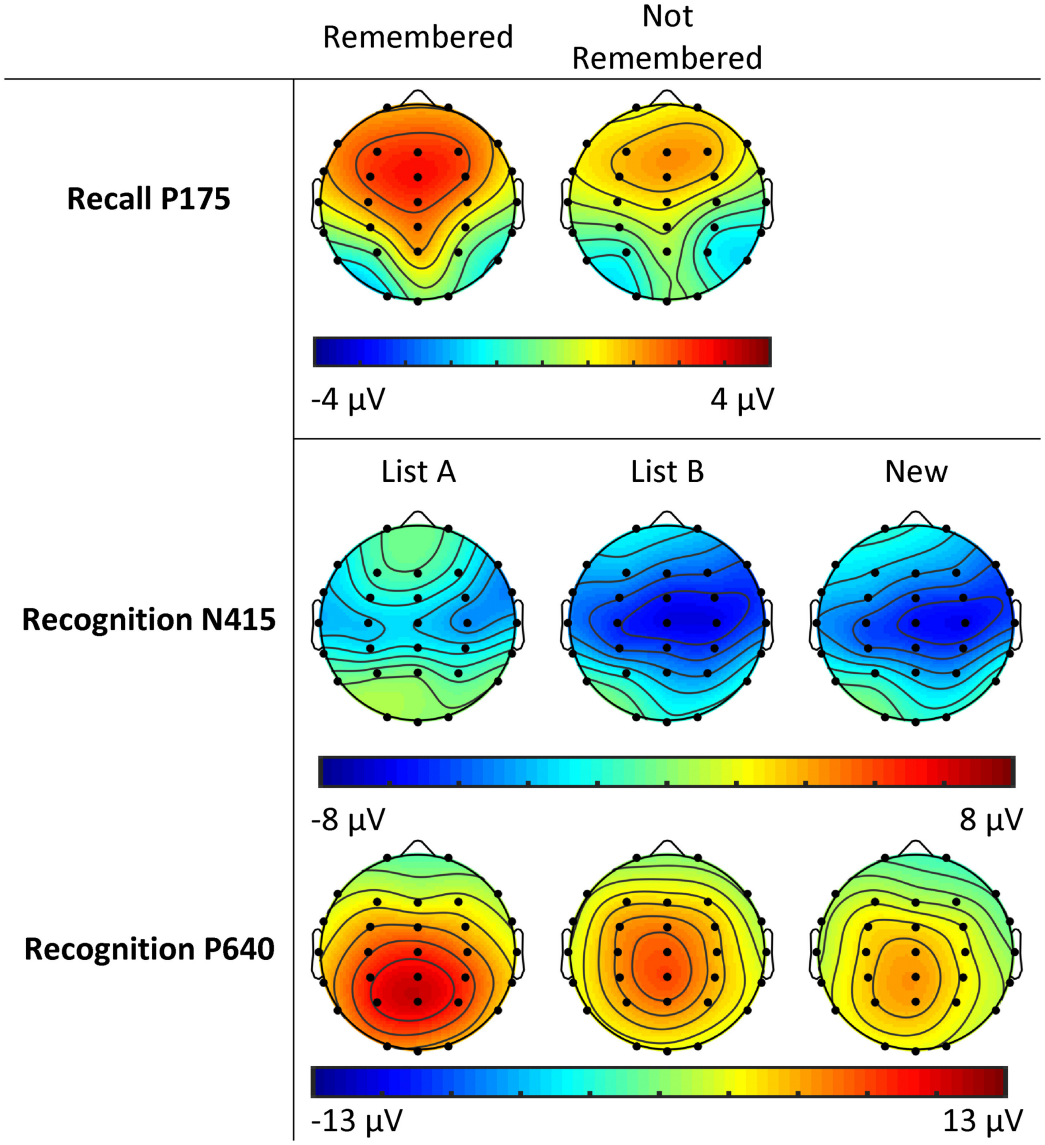
Topographic plots of activity across sites and conditions for P175 in the Recall phase, and N415 and P640 in the Recognition phase for adolescent males (Study 1).

Figure 2 (left) shows the grand mean waveforms for ERPs in the Recall phase for Remembered and Not Remembered words. The PCA-identified P175 was larger at frontal than parietal sites (*F* = 18.17, *p* < 0.001; all *df* = 1.32), and had a tendency to larger amplitudes at central than frontal/parietal sites (*F* = 3.95, *p* = 0.056; see Figure 3 for topographic plots of activity across sites). It was also larger in the midline than hemispheres (*F* = 37.50, *p* < 0.001). At frontal sites, the midline > hemispheres effect was reduced compared to the effect at parietal sites (*F* = 13.95, *p* = 0.001), while at central sites a left > right effect was observed, which was reversed and reduced at frontal/parietal sites (*F* = 9.03, *p* = 0.005).

There was a Type main effect (*F* = 12.37, *p* = 0.001), with larger amplitudes for Remembered than Not Remembered words, particularly at central compared to frontal/parietal sites (*F* = 2.99, *p* = 0.093). Remembered words showed a small left > right effect at frontal sites, and a larger right > left effect at parietal sites, while Not Remembered words showed a small right > left effect frontally, and a larger left > right effect at parietal sites (*F* = 4.68, *p* = 0.038). Further, the midline > hemispheres effect was equal in magnitude for Remembered and Not Remembered words at frontal sites, but was larger for Remembered than Not Remembered words at parietal sites (*F* = 5.60, *p* = 0.024).

For completeness, the analyses of the P210 and P260 components (neither of which showed significant Remembered > Not Remembered effects) are included in Supplementary Material, as well as analyses of the later N440 and P630 components identified by the PCA of the Recall ERPs.

### Recognition ERPs

Figure 2 (right) shows the grand mean waveforms for ERPs in the Recognition phase for List A, List B and New words. Despite relatively few trials being included in each participant's average, the grand mean ERPs nonetheless present component morphology in line with expectations. Again, a clear N1-P2 complex can be seen, followed by a frontal negativity peaking around 400 ms and appearing similar in amplitude for List B and New words, followed by a larger parietal late positivity peaking around 550 ms, largest for List A words.

### N415

Topographic plots of N415 activity are presented in Figure 3. The N415 was more negative at central than frontal/parietal sites (*F* = 57.77, *p* < 0.001; *df* = 1, 31 for this and P640 topographic analyses), and more negative in the right than left hemisphere (*F* = 5.59, *p* = 0.025). For New words vs. previously seen words, the N415 tended to be more negative for New than List A/B words (*F* = 3.34, *p* = 0.077). New words were associated with a midline > hemispheres effect, while the opposite was observed for previously seen words (*F* = 5.98, *p* = 0.020). Comparing between previously seen words, List B words showed greater negativity than List A words (*F* = 14.58, *p* = 0.001). List A words showed reduced midline amplitude relative to the hemispheres, while List B words showed greater midline than hemispheric amplitude (*F* = 29.05, *p* < 0.001). List A words showed a slightly larger right > left effect at central compared to frontal/parietal sites, while List B words showed a reduced effect at central relative to frontal/parietal sites (*F* = 4.35, *p* = 0.045).

### P640

The P640 was more positive at parietal than frontal sites (*F* = 33.00, *p* < 0.001) and at central than frontal/parietal sites (*F* = 20.23, *p* < 0.001; see Figure 3). Positivity was greater on the left than right (*F* = 5.51, *p* = 0.025), and greater still at midline sites (*F* = 16.54, *p* < 0.001). The midline > hemispheres effect was greater at parietal than frontal sites (*F* = 29.25, *p* < 0.001), and greater still at central sites (*F* = 24.74, *p* < 0.001).

In comparisons of List A with Other (List B and New) words, a type main effect was apparent (*F* = 6.82, *p* = 0.014), with greater positivity for List A words. This was particularly the case at parietal compared to frontal sites (*F* = 48.08, *p* < 0.001). Additionally, List A words displayed a midline > hemispheres effect parietally, and a much smaller, and reversed effect frontally, while Other words were associated with midline > hemispheres effect at both frontal and parietal sites (*F* = 15.80, *p* < 0.001).

In comparisons of List B with New words, greater positivity was observed for List B words (*F* = 11.90, *p* = 0.002). The topography of this differed: List B words were associated with a somewhat smaller parietal > frontal gradient than New words (*F* = 3.65, *p* = 0.066). The left > right effect was somewhat stronger for New than List B words (*F* = 3.16, *p* = 0.085).

On the whole, we observed the ERP components that we expected based on research using other memory tasks, with typical topographies and differences in amplitude according to trial type (words which were Remembered vs. Not Remembered, in the Recall phase, and for List A, List B and New words in the Recognition phase). This suggests that PCA can be used to identify ERP components during the RAVLT; our study is the first to attempt this with healthy control participants, let alone substance-using groups (see Supplementary Materials). The P175 showed a Remembered > Not Remembered main effect, with a frontal maximum as expected (e.g., Mangels et al., 2001). Similarly, N415 and P640 in the recognition phase showed the expected frontocentral and centroparietal maxima, respectively. N415 was larger for New than familiar (List A and List B) words, and larger for List B than List A words, consistent with a component reflecting (un)familiarity ( e.g., Rugg et al., 1998; Curran, 2000), while P640 was largest for List A words, consistent with correct recollection of the source of the word (Rugg et al., 1998; Curran, 2000), and larger for List B than New words (although the latter effect is of course confounded with familiarity). In summary, this pilot study provides proof of concept that meaningful ERP components associated with recall and recognition can be extracted using PCA techniques in a modified version of the RAVLT, and that these behave in a manner predictable from other research.

However, in this pilot study with appropriately small sample sizes, we were underpowered to detect group differences associated with alcohol and/or cannabis use (reported in Supplementary Materials), particularly since we recruited relatively light drinkers and cannabis users, compared to our previous studies of heavier users where greater deficits might be expected (e.g., Solowij et al., 2011). In the second study, we report the results of a separately conducted examination of a larger sample of young adults. For this study, we collected more detailed information about use of alcohol, cannabis, and other drugs, with eligibility criteria requiring slightly heavier use, larger samples including both male and female participants, and recorded EEG from a denser scalp montage to increase the information available for PCA.

## Study 2

## Methods

### Participants

Participants were 104 young adults (aged between 18 years and 21 years 11 months), who were recruited into three groups based on their reported use of alcohol and cannabis. The “Cannabis Users” (CU) group (9 females, 11 males) used cannabis regularly (at least twice a month in the past year). The “Heavy Drinkers” (HD) group (16 females, 23 males) engaged in heavy drinking (four or more Australian standard drinks, equal to 40 g alcohol, on one occasion) regularly (at least monthly in the past year), but used cannabis less than twice a month over the past year (including irregular/occasional use, and never having used cannabis). Lastly, the “Drug-Naive Controls” (DNC) group (20 females, 25 males) neither used cannabis regularly (less than twice a month over the past year, including never) nor engaged in heavy drinking regularly (less often than once a month over the past year, including irregular heavy drinkers, those who never engaged in heavy drinking, and those who did not drink any alcohol).

Participants were recruited via posters displayed on the university campus and via participant referral, and were excluded if they had ever had an epileptic seizure, a serious head injury or period of unconsciousness, uncorrected hearing or vision problems, or regular (at least twice a month) use of other drugs. Additionally, participants reported no use of medication other than contraception or antibiotics. Participants were screened for a history of psychiatric illness: 3 participants in each group (2 female DNC, 2 female HD and 3 female CU) reported depression and/or anxiety; all other participants reported no personal history of psychiatric illness. We did not assess or screen for a family history of psychiatric illness, including substance abuse. All participants gave written informed consent, and the protocol was approved by the University of New South Wales Human Research Ethics Committee before data collection began in an EEG laboratory at the University of New South Wales. Our sample represented the Australian population, in which over half of those aged 18-21 years regularly drink heavily (that is, consume more than four standard drinks, equivalent to 40 g alcohol, at least once a month; AIHW, 2011), while approximately 10% of 18–29 year olds use cannabis at least once a month (AIHW, 2011).

### Procedures

The experimenter showed the participant the lab and recording equipment and described the experimental protocol before written informed consent was obtained. Participants then completed a short demographics questionnaire and modified versions of the Alcohol Use Disorders Identification Test (AUDIT, Saunders et al., 1993) and the Drug Use Disorders Identification Test-Extended (DUDIT-E, Berman et al., 2007). Question 3 of the AUDIT was modified from “How often do you have six or more standard drinks on one occasion?” to “four or more standard drinks” to reflect Australian alcohol consumption guidelines (NHMRC, 2009). Participants were requested to reference a standard drinks guide provided while they completed this section. Only the first section of the DUDIT-E was administered, and was used to screen participants for eligibility to the study. That section assesses the frequency of use of a range of drug classes other than alcohol, with the options: Never (score = 0), Tried it once or more (1), Once a month or less often (2), 2–4 times a month (3), 2–3 times a week (4), 4 times a week or more (5). Twenty-nine DNC and 12 HD participants had a total score of zero (CU by definition scored at least 3), and no participant in this study scored more than 2 for any drug class (except tobacco and cannabis; this was an exclusion criterion of the study). Use of tobacco does not contribute to the total score. Table 1 shows the demographic characteristics of the participants included in the study.

**Table 1:** Demographic information for males and females in each group in the sample of young adults (Study 2).

	**Drug-Naive Controls (DNC)**				**Heavy drinkers (HD)**				**Cannabis users (CU)**			
	**Females (*****n*** = **20)**		**Males (*****n*** = **25)**		**Females (*****n*** = **16)**		**Males (*****n*** = **23)**		**Females (*****n*** = **9)**		**Males (*****n*** = **11)**	
	**Mean**	**SD**	**Mean**	**SD**	**Mean**	**SD**	**Mean**	**SD**	**Mean**	**SD**	**Mean**	**SD**
Age (years)	19.9	1.2	20.0	1.1	20.0	1.2	19.7	1.2	20.1	1.2	20.5	1.2
% Daily smokers	0.0		0.0		0.0		13.0		11.1		27.3	
% Right handed	90.0		100.0		81.3		78.3		77.8		90.9	
AUDIT score#	3.1	2.9	2.9	3.0	9.5	3.3	9.1	2.6	9.3	4.6	11.4	4.6
Lifetime standard drinks (log units)^†^	1.4	1.2	1.5	0.9	2.7	0.5	2.6	0.4	2.8	0.5	3.0	0.4
Lifetime standard drinks (mean and CI)^†^	25.2	(7.8–81.4)	31.6	(14.2–70.0)	470.4	(254.4–869.8)	391.0	(267.6–571.3)	624.6	(314.4–1240.8)	1, 029.2	(581.9–1,820.1)
Age of onset of regular drinking	18.5	1.6	18.0	1.7	17.5	1.6	17.3	1.3	16.3	1.2	16.6	1.0
Duration of regular drinking	1.4	1.5	2.0	1.7	2.5	1.6	2.4	1.4	3.8	1.7	3.9	1.1
DUDIT-E score	0.7	1.3	0.6	0.8	1.4	1.5	2.2	2.1	6.7	2.0	7.5	3.8
DUDIT-E range	0–4		0–2		0–4		0–9		3–9		3–15	
Age first regular cannabis use^*^									17.0	1.2	17.4	1.2
Duration regular cannabis use, years^*^									2.1	1.5	2.6	1.1
Frequency of cannabis use (days/month), last 6 months, median and range^*^									4.2	(1.8–18.2)	12.9	(2.0–30.0)

# *AUDIT scores are useful only for internal comparison between groups, due to the alteration to Question 3 as described in the text*.

† *Due to non-normal distributions, lifetime standard drinks were converted to log scores prior to statistical analysis; the mean and SD reported are in log units, while the numbers in brackets below give the inverse log of the mean, and the confidence interval calculated for that mean, in units of standard drinks*.

* *n = 7 females, 10 males; data were not recorded for the first three cannabis user participants*.

All participants also underwent structured interviews assessing lifetime alcohol use and lifetime cannabis use using a modified version of the Lifetime Drinking History interview (Skinner, 1977). This assesses the frequency and quantity of alcohol consumption in relatively homogenous phases from the age of onset of regular drinking (one standard drink per month), and can be used to assess the number of standard drinks consumed in the participant‘s lifetime [because these scores are non-normally distributed, statistical analysis is performed on the log (base 10) of total consumption+1, to avoid taking the log of zero]. Participants referred to the standard drinks guide during the alcohol section of the interview. For participants who had never consumed one standard drink per month, the age of onset was entered as the participant's age on the day of testing, and the duration (years) of regular drinking was entered as zero. The cannabis section was used to calculate the age of first regular use, the duration of regular use, and frequency of use in the 6 months prior to testing for the cannabis user group.

### EEG recording and analysis

**Figure 4:**
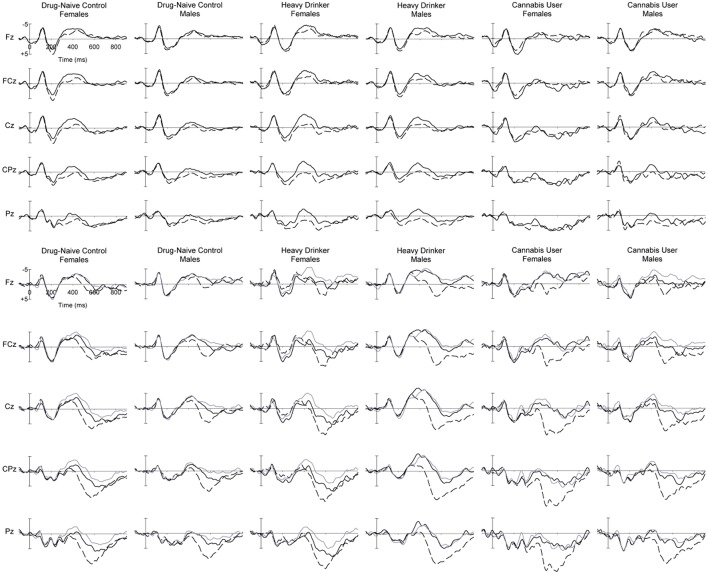
Grand mean ERPs at midline sites for (top panel) words which were later Remembered (dashed) and Not Remembered (solid) words in the Recall phase, and (bottom panel) List A (dashed), List B (black solid) and New (gray solid) words in the Recognition phase, for female and male young adults (Study 2) in the DNC, HD and CU groups.

The RAVLT task was completed and scored as described for Study 1. Continuous monopolar EEG was recorded from 60 scalp sites using an elasticised cap with tin electrodes. Additional tin cup electrodes recorded activity from the left and right mastoid as well as vertical and horizontal EOG. All electrodes were referenced to an electrode on the tip of the nose, grounded midway between FPz and Fz. Electrode impedances were below 5 kΩ. Signals were recorded DC to 200 Hz, amplified 10 times, and sampled at 1,000 Hz using NeuroScan recording software and hardware (Synamps 2). EEG data was re-referenced offline to linked mastoids before filtering, eye movement correction, interpolating, epoching, baselining, artifact rejection and averaging proceeded as described for Study 1. One female HD participant had exceptionally noisy mastoid channels in both the recall and recognition EEG files, and her data were excluded from all ERP analyses (but included in behavioral measures). The EEG file for the recognition phase was lost for one male HD; however, his behavioral performance for that phase could be retrieved from the Presentation log file. The grand mean waveforms for the Recall and Recognition phases of the experiment are displayed in Figure 4.

### Data reduction

PCA for the ERP data proceeded as described in Study 1. The PCA on Remembered and Not Remembered trials had 12,360 cases (103 participants × 2 conditions × 60 sites), and factor extraction and rotation was restricted to 16 factors on the basis of Horn's Parallel Test (Horn, 1965), together accounting for 93.73% of variance. Two positive factors were identified in the P2 time range. Only one of these, labeled P185 (Factor 5 peaking at 185 ms, maximal at FC1, explaining 6.29% of unique variance) displayed a Remembered > Not Remembered effect. Analyses of the other P2-like factor (Factor 4), as well as factors peaking at 380 ms (Factor 2) and 535 ms (Factor 1) are described in Supplementary Material.

The PCA on List A, List B and New words had 18,360 cases (102 participants × 3 conditions × 60 sites); factor extraction and rotation was restricted to 14 factors, together explaining 93.46% of variance. Factor 1 was labeled P540 (peaking at 540 ms, maximal at P1, explaining 25.08% of variance), and was identified as reflecting the classical parietal Old/New effect, while Factor 3, labeled N340 (peaking at 340 ms, maximal at Cz, explaining 19.53% of variance) was identified as reflecting the frontocentral N400 effect.

### Statistical analysis

Statistical analysis for demographic and behavioral measures proceeded as described for Study 1 except with the additional factors Sex and Group being included in MANOVAs for behavioral measures. We included sex as a factor in our analyses because women tend to outperform men on verbal memory tasks (e.g., Andreano and Cahill, 2009; Carstairs et al., 2012), because there is growing evidence that males and females may be differently susceptible to the long-term cognitive effects of chronic alcohol and cannabis misuse (e.g., Pope and Yurgelun-Todd, 1996; Townshend and Duka, 2005; Crane et al., 2013), and because the inclusion of women in the young adult but not adolescent sample may contribute to differences between the study results. Contrasts on the Group factor (for this and all other analyses) separately compared the performance of DNC with HD, and HD with CU. These group comparisons were selected because alcohol consumption by the CU group was similar to that in the HD group (see results). Thus the DNC vs. HD comparison assesses the effect of heavy drinking, while the CU vs. HD comparison assesses the effect of cannabis use while controlling for heavy drinking; although we allow that there could be interactive effects of alcohol and cannabis, examination of these is beyond the scope of this study.

A two-step approach was taken with analyses of ERP data, to accurately describe the topography of the PCA-identified components, and to assess the important Group and Sex main effects and interactions. First, as in Study 1, peak component amplitudes from the sites F3, Fz, F4, C3, Cz, C4, P3, Pz, and P4 were each assessed with three-way MANOVAs with factors Lateral (left/midline/right), Sagittal (frontal/central/parietal) and Type (for the recall phase: Remembered, Not Remembered; for the recognition phase: List A, List B, New), with contrasts as described above.

In the second step, the average activity was calculated from a number of sites identified via the above step as the regions of maximum amplitude, and these single variables (one for each component) were entered into separate Type × Group × Sex MANOVAs. Contrasts on the Group and Type factors were as mentioned above. An alpha level of 0.05 was adopted throughout Study 2, although we report limited effects approaching significance where they indicate the possibility of group differences, and also report effect sizes (Cohen's *d*) where appropriate. In all cases, a negative effect size represents poorer performance in the HD than DNC group, or in the CU than HD group.

## Results and discussion

### Demographics

The groups were well matched for age, with no significant effects of group or sex (all *p* > 0.175; for this section *df* = 1.98 unless otherwise reported). Within each group, the proportion of right-handed participants was not significantly different between males and females (all *p* > 0.106). Within each group, the proportion of daily tobacco use was equal between males and females (all *p* > 0.133). Greater AUDIT scores were observed for HD relative to DNC (*F* = 74.36, *p* < 0.001), but were equal for HD relative to CU (*F* = 1.33, *p* = 0.251), with no main effects or interactions with sex (all *p* > 0.184). (Log) lifetime drinks were significantly greater for HD relative to DNC (*F* = 50.08, *p* < 0.001), but were equal for CU relative to HD (*F* = 1.68, *p* = 0.197), with no main effects or interactions with sex (all *p* > 0.480). Age of onset of regular drinking was significantly younger for HD relative to DNC (*F* = 7.17, *p* = 0.009), and younger still for CU relative to HD (*F* = 5.41, *p* = 0.022), with no main effects or interactions with sex (all *p* > 0.599). Consistent with this, the duration of regular drinking was longer for HD relative to DNC (*F* = 4.77, *p* = 0.031), and for CU relative to HD (*F* = 10.94, *p* = 0.001), with no sex main effects or interactions (all *p* > 0.287). DUDIT scores were significantly greater for the HD compared to DNC group (*F* = 7.89, *p* = 0.006) and for the CU compared to HD group (*F* = 102.06, *p* < 0.001), with no sex main effects or interactions (all *p* > 0.219). The increase for the HD relative to DNC group was mainly due to a greater incidence of experimentation with cannabis; when cannabis use frequency was excluded from the total score, HD scores were not significantly different to DNC (*F* = 2.81, *p* = 0.097). The increase for the CU relative to HD group was due mostly to the increased cannabis use score but also partly due to a greater incidence of experimentation with other drugs; when cannabis use frequency was excluded from the total score, CU still scored significantly higher than HD (*F* = 29.91, *p* < 0.001). For the CU group, there were no sex differences for age of first regular use [*F*_(1, 15)_ = 0.28, *p* = 0.606], duration of regular use [*F*_(1, 15)_ = 0.60, *p* = 0.451], or frequency of use in the past 6 months (*p* = 0.193). Seven of the female CU and 10 of the male CU engaged in heavy drinking at least monthly (χ^2^ = 3.65, *p* = 0.301).

In summary, we recruited samples of young adults which were generally comparable, but differed as expected on the substance use measures. However, while it was our intention to match the CU and HD groups for alcohol use, in order to examine the effect of cannabis use after controlling for alcohol use, we note that the CU group showed an earlier onset and longer duration of regular alcohol use, despite similar consumption overall. Therefore, it is possible that this early alcohol exposure, rather than cannabis use *per se*, may be responsible for any group differences observed between CU and HD groups. Furthermore, since we used an Australian definition of binge drinking (consumption of 40 g of alcohol on one occasion; NHMRC, 2009), the ability to compare our sample with others using different definitions of binge/heavy drinking (e.g., NIAAA, 2004) is somewhat limited. However, we point out that there was considerable variation above the minimum quantity/frequency criterion for entry to the study, and that it seems likely that similar outcomes would be observed however the groups were constructed, as in the literature concerning inhibitory control among heavy drinkers reviewed in Smith et al. (2014). Lastly, we did not assess or control for the presence of a family history of psychiatric illness, including substance abuse; this is an important predictor of cognitive dysfunction (e.g., Acheson et al., 2009, 2014), and should be screened for in future research.

### Behavioral performance

Table 2 shows performance measures for each group and sex; for the analyses reported here *df* = 1.98. Across groups, there were highly significant increases in the number of words remembered over Trials I-V (linear trend *F* = 1096.83, *p* < 0.001; quadratic trend *F* = 158.70, *p* < 0.001), indicating learning across trials. There was a non-significant trend to a greater linear increase over Trials I-V for the HD compared to the CU group (*F* = 3.44, *p* = 0.067). Further, a Group × Sex × Trial interaction approached significance (*F* = 3.7, *p* = 0.056, linear trend), such that male DNC and HD had similar learning over trials, while female HD actually had greater learning over trials than female DNC.

**Table 2:** Behavioural performance for males and females in the Drug-Naïve Controls (DNC), Heavy Drinker (HD) and Cannabis User (CU) groups in the sample of young adults (Study 2).

	**DNC**				**HD**				**CU**			
	**Females (*****n*** = **20)**		**Males (*****n*** = **25)**		**Females (*****n*** = **16)**		**Males (*****n*** = **23)**		**Females (*****n*** = **9)**		**Males (*****n*** = **11)**	
	**Mean**	**SD**	**Mean**	**SD**	**Mean**	**SD**	**Mean**	**SD**	**Mean**	**SD**	**Mean**	**SD**
**RECALL PHASE PERFORMANCE**												
Trial I	7.1	1.5	6.4	1.7	6.4	1.5	6.6	1.6	7.2	1.7	7.4	2.5
Trial II	10.7	1.8	10.1	1.5	10.0	2.0	9.6	1.9	9.9	2.1	10.2	3.0
Trial III	12.0	1.9	11.6	1.4	12.1	1.7	11.0	2.0	12.1	1.5	12.0	2.6
Trial IV	13.0	1.4	12.5	1.2	13.3	1.7	11.7	1.9	12.1	1.2	12.2	2.0
Trial V	13.2	1.3	12.8	1.5	13.6	1.2	12.8	1.9	12.9	1.1	13.5	1.0
Trial B	6.0	1.5	6.2	1.6	6.5	1.9	5.6	1.8	6.7	2.0	5.7	2.1
Trial VI	12.4	1.7	11.7	2.1	12.3	2.3	11.2	2.1	11.8	2.0	12.2	2.6
Trial VII	12.5	2.0	11.9	1.5	12.1	2.4	10.1	2.6	11.9	1.5	11.5	3.0
Total words recalled (Trials I-V)	55.8	5.7	53.5	5.2	55.3	6.0	51.8	7.6	54.2	6.4	55.2	10.3
Learning rate (V minus I)	6.1	1.7	6.4	1.9	7.1	1.3	6.3	2.0	5.7	1.2	6.1	1.8
Proactive interference (I minus B)	1.1	1.6	0.2	2.0	−0.1	2.4	1.0	1.8	0.6	2.1	1.6	2.1
Retroactive interference (V minus VI)	0.8	1.6	1.1	1.6	1.3	1.7	1.7	1.5	1.1	1.4	1.3	1.9
Forgetting (V minus VII)	0.7	2.2	0.9	1.4	1.5	1.9	2.7	1.8	1.0	1.2	1.9	2.2
**RECOGNITION PHASE PERFORMANCE**												
List A accuracy (number correct/15)	14.3	1.0	14.1	1.2	13.8	1.8	14.2	1.2	14.0	1.3	14.2	1.1
List B accuracy (number correct/15)	14.2	1.1	14.2	1.0	14.4	1.0	14.2	1.3	13.8	1.2	13.8	1.2
New accuracy (number correct/20)	18.9	1.1	18.9	1.3	19.3	0.7	18.8	1.4	19.0	1.0	18.5	1.9
List A RT (ms)	891.3	222.3	948.5	243.2	811.4	207.7	907.1	271.5	923.9	338.1	869.9	237.8
List B RT (ms)	802.7	153.2	939.1	281.3	824.7	293.1	912.0	218.7	937.6	325.7	890.2	204.8
New RT (ms)	864.3	232.9	977.2	250.9	792.5	178.1	973.6	227.4	883.0	284.2	915.2	283.5
**PEAK COMPONENT AMPLITUDES**												
Remembered P185^†^	4.1	3.0	3.4	2.3	2.5	2.0	3.2	2.4	2.7	1.6	3.3	2.2
Not Remembered P185^†^	3.2	3.4	2.7	2.5	2.4	1.4	2.6	2.7	2.8	2.4	2.5	3.1
List A N340^†^^*^	−5.7	4.7	−3.7	4.5	−5.9	6.2	−6.7	4.9	−1.2	7.1	−4.5	4.4
List B N340^†^^*^	−5.1	4.8	−3.5	4.3	−3.9	6.5	−6.9	4.4	−0.4	9.2	−3.8	4.2
New N340^†^^*^	−5.6	3.8	−2.6	3.5	−5.0	6.0	−4.9	3.7	−1.3	6.0	−4.0	3.2
List A P540^†^^*^	9.5	3.9	7.2	3.8	11.0	5.7	10.0	4.5	11.9	5.0	8.0	4.0
List B P540^†^^*^	4.1	2.7	1.5	4.4	5.6	4.3	3.4	4.4	3.2	4.4	2.4	3.2
New P540^†^^*^	2.7	3.3	0.9	2.9	3.5	2.8	2.3	3.0	4.3	3.0	1.5	1.8

† N = 15 for female HD

* N = 22 for male HD

Participants remembered fewer words for Trial B than for Trial I (*F* = 12.48, *p* = 0.001), indicating proactive interference. Furthermore, a Group × Sex × Trial effect reached significance (*F* = 4.55, *p* = 0.035), such that for females, proactive interference was greater for DNC than HD (*d* = 0.548), but the opposite was true for males (*d* = −0.402). There were no significant differences between HD and CU groups (all *p* > 0.236, effect size across sexes *d* = −0.277). Participants also remembered significantly fewer words for Trial VI compared to Trial V (*F* = 51.30, *p* < 0.001), indicating retroactive interference. There were no main effects or interactions involving group or sex for retroactive interference (all *p* > 0.125).

Participants remembered significantly fewer words for Trial VII than for Trial V (*F* = 58.54, *p* < 0.001), indicating forgetting after a 20 min delay. Greater forgetting was apparent in HD compared to DNC (*F* = 10.61, *p* = 0.002, *d* = −0.766), and in males compared to females (*F* = 4.27, *p* = 0.041, *d* = 0.404). Forgetting was equivalent for HD and CU (*p* > 0.190, *d* = 0.388).

Regarding recognition performance, there were no significant differences between sexes or groups for accuracy to List A (all *p* > 0.293), List B (all *p* > 0.093) and New words (all *p* > 0.228). There was a significant sex difference in the RT to List A vs. New words (*F* = 3.94, *p* = 0.050), such that females were faster to respond to New than List A words, while males were slower to New than List A words. There were no interactions involving group.

Thus, it appears the performance of our sample is relatively normal and demonstrates the expected changes over trials, although, similar to Study 1, performance is slightly poorer than published norms (e.g., Carstairs et al., 2012). Overall, females did slightly (but non-significantly) better than males, consistent with previous reports of a slight verbal memory advantage for females (e.g., Andreano and Cahill, 2009; Carstairs et al., 2012). Further, there were tendencies for increased learning over trials in HD than CU, and in female HD than female DNC, although neither of these reached significance. However, the substantially greater forgetting after a 20 min delay in HD bears some discussion: HD lost an average of 2.2 words (1.5 for females and 2.7 for males). For comparison, females typically forget 1.0 word on average, while males forget 1.7 words (Carstairs et al., 2012); the increased forgetting is unlikely to be due to the modifications to RAVLT delivery in our study, since our DNC actually forgot fewer words than the normative samples (our female DNC = 0.7 words, male DNC = 0.9 words). Thus, our study highlights particular problems with forgetting/delayed recall in heavy drinkers, an effect which is reported sometimes (e.g., Waugh et al., 1989; Brown et al., 2000; Pitel et al., 2009) but not always (e.g., Kokavec and Crowe, 1999; Parada et al., 2011; Sanhueza et al., 2011; Solowij et al., 2011; Mota et al., 2013; Sneider et al., 2013; Winward et al., 2014).

The lack of significant deficits in CU is clearly in contrast to many previous studies which have reported significant memory deficits in cannabis users (e.g., Yücel et al., 2008; Solowij et al., 2011). We reference those two studies in particular because they utilized a similar approach as here, by controlling for alcohol use, which is itself associated with learning and memory deficits. Yücel et al. matched controls and cannabis users for alcohol use, while Solowij et al. recruited DNC, HD, and CU groups and reported all pairwise comparisons. It is unclear why we do not observe deficits associated with cannabis use (after controlling for alcohol use): it is not the case that, due to the smaller sample size of the current study, our statistical power was too low to detect cannabis-related deficits. Rather, the obvious deficit for CU relative to HD in Solowij et al. (e.g., total words recalled Cohen's *d* = −0.748) was absent in our study, with slightly *more* words recalled for CU than HD (*d* = 0.199). A more likely explanation concerns dose-dependent and possibly age effects: our sample consists of considerably lighter cannabis users than Yücel et al., and though it is more similar to the sample in Solowij et al., in terms of recruitment criteria, alcohol use (AUDIT scores), and duration and age of onset of regular cannabis use, the Solowij et al. (2011) sample were somewhat younger than ours (mean 18 vs. 20 years), as well as being more frequent users and possibly heavier users per occasion. Thus, it is possible that dose-dependent and/or age effects might explain our lack of significant memory disruption in cannabis users.

### Recall ERPs

**Figure 5:**
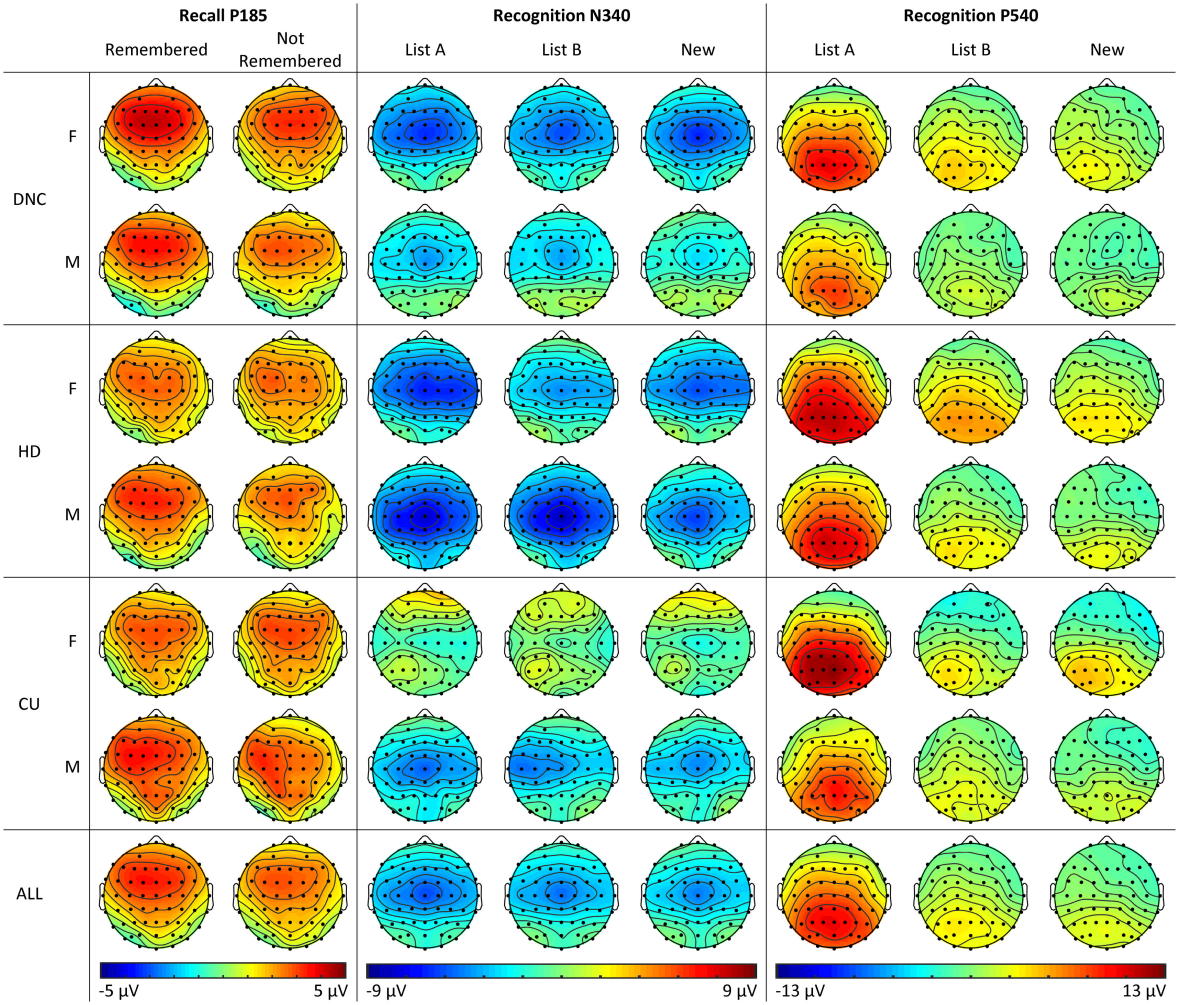
Topographic plots of activity across sites, groups and conditions for P185 in the Recall phase, and N340 and P540 in the Recognition phase for female and male young adults (Study 2).

Grand mean ERPs in the Recall phase of the experiment can be observed in Figure 4 (top), while topographic maps of activity can be seen in Figure 5. Generally similar waveform morphology is observed, compared to the adolescents in Figure 2. Again, a clear N1-P2 complex is observed, with an appearance of larger P2 amplitudes for Remembered than Not Remembered words, followed by a frontocentral negative wave around 400 ms, appearing larger for Not Remembered words.

Statistical analyses confirmed these observations: the PCA-identified P185 showed a frontal > parietal effect (*F* = 27.59, *p* < 0.001), and also larger amplitudes centrally than at frontal/parietal sites (*F* = 40.40, *p* < 0.001, *df* = 1.102). It was also larger in the midline than hemispheres (*F* = 35.87, *p* < 0.001). At frontal sites, a left > right effect was observed, which was reversed at parietal sites (*F* = 4.21, *p* = 0.043). Further, the midline > hemispheres effect was reduced at frontal compared to parietal sites (*F* = 8.81, *p* = 0.004).

P185 was marginally larger for Remembered than Not Remembered words (*F* = 3.88, *p* = 0.051), particularly at frontal sites (*F* = 5.32, *p* = 0.023). The midline > hemispheres effect was also larger for Remembered words (*F* = 4.57, *p* = 0.035). Remembered words showed a larger reversal of the parietal to frontal laterality effect than Not Remembered words (*F* = 5.45, *p* = 0.022).

The average of sites F1, Fz, F2, FC1, FCz, and FC2 were entered into the second MANOVA; means and SDs are presented in Table 2 for each condition and group. The Type main effect was now significant (*F* = 5.43, *p* = 0.022, *df* = 1.97), again with larger amplitudes for Remembered words. However, no other main effects or interactions were significant (magnitude of Remembered > Not Remembered effect, difference between groups DNC vs. HD: *F* = 1.19, *p* = 0.278, *d* = −0.216; HD vs. CU: *F* = 0.00, *p* = 0.990, *d* = −0.007).

### Recognition ERPs

Figure 4 (bottom) shows the grand mean waveforms for ERPs in the Recognition phase for List A, List B and New words for young adults. Again, the waveform morphology was similar to the adolescent group, and in line with expectations. A clear N1-P2 complex can be seen, followed by a frontal negativity peaking around 450 ms and appearing similar in amplitude for List B and New words for most groups, followed by a larger parietal late positivity peaking around 550 ms, largest for List A words. Statistical analyses of the PCA-identified N340 and P540 components are presented next.

### N340

All topographic analyses for N340 and P540 have *df* = 1.101. N340 showed a frontal > parietal effect (*F* = 13.14, *p* < 0.001), and a central > frontal/parietal effect (*F* = 252.30, *p* < 0.001; see Figure 5). Amplitudes were more negative in the midline than hemispheres (*F* = 53.03, *p* < 0.001), particularly at central compared to frontal/parietal sites (*F* = 34.25, *p* < 0.001).

N340 was also more negative for words seen before (List A and List B) compared to New words (*F* = 6.70, *p* = 0.011). The midline > hemispheres effect was stronger for New than old words (*F* = 4.98, *p* = 0.028), particularly at parietal relative to frontal sites (*F* = 8.74, *p* = 0.004). For List A words, there was a small right > left effect frontally, but similar amplitudes parietally, while for List B words, there was a small left > right effect frontally, and a larger right > left effect parietally (*F* = 5.18, *p* = 0.025).

The average of sites FC1, FCz, FC2, C1, Cz, and C2 were entered into the second ANOVA, with *df* = 1.96 for both N340 and P540; means and SDs are presented in Table 2 for each condition and group. There were no significant effects for List A vs. List B words. For New vs. old words, males showed larger amplitudes to old words while females showed larger amplitudes to new words (*F* = 6.24, *p* = 0.014). The N340 was smaller overall in CU relative to HD (*F* = 5.60, *p* = 0.020, *d* = −0.586).

### P540

The P540 was more positive at parietal than frontal sites (*F* = 143.39, *p* < 0.001). Greater amplitudes were observed on the left than right (*F* = 24.59, *p* < 0.001) and in the midline compared to the hemispheres (*F* = 19.79, *p* < 0.001). This midline > hemispheres effect was stronger at parietal than frontal sites (*F* = 17.23, *p* < 0.001), and at central compared to frontal/parietal sites (*F* = 5.56, *p* = 0.020).

P540 was much larger for List A than Other words (*F* = 268.97, *p* < 0.001), particularly at parietal compared to frontal sites (*F* = 52.27, *p* < 0.001) and at central compared to frontal/parietal sites (*F* = 17.20, *p* < 0.001). Also, the midline > hemispheres was greater for List A than Other words (*F* = 27.24, *p* < 0.001). A slight left parietal dominance was observed for List A words, in line with previous research, although this did not reach significance (*F* = 3.44, *p* = 0.067): for List A words, the left > right effect was slightly greater at parietal than frontal sites, while for Other words, the effect was slightly greater frontally. The parietal > frontal × midline > hemispheres effect was greater for List A than Other words (*F* = 6.31, *p* = 0.014), as was the central > frontal/parietal × midline > hemispheres effect (*F* = 5.52, *p* = 0.021).

List B words were associated with greater positivity than New words (*F* = 12.22, *p* = 0.001), particularly at central sites relative to frontal/parietal (*F* = 21.04, *p* < 0.001). For List B words, a left > right effect was similar in magnitude frontally and parietally, while for New words, the left > right effect was much larger frontally (*F* = 4.65, *p* = 0.033).

The average activity from sites P3, P1, Pz, PO3, and POz were entered into the second MANOVA; means and SDs are presented in Table 2. A main effect of sex was significant (*F* = 9.42, *p* = 0.003), with larger P540 amplitudes in women than men, and amplitudes were also greater in HD relative to DNC (*F* = 5.54, *p* = 0.021, *d* = 0.503). P540 was substantially larger for List A vs. Other words (*F* = 320.77, *p* < 0.001), but no interactions with group or sex were significant. P540 was also larger for List B compared to New words (*F* = 6.06, *p* = 0.016). There was a tendency for a reduced List B > New effect for CU compared to HD, but this effect did not reach significance (*F* = 3.44, *p* = 0.067, *d* = −0.432). This effect did not differ between HD and DNC (*F* = 0.73, *p* = 0.395, *d* = 0.173).

Within-subject ERP results for the Recall P185 and the Recognition P540 were broadly in line with expectations for topographic and condition effects, and similar to Study 1. Additionally, the recognition N340 showed the expected frontocentral midline maximum. However, a sex difference was observed for the N340: males showed an unexpected increase in N340 amplitude to List A words, opposite to the females in this study, and reported in previous research (e.g., Rugg et al., 1998; Curran, 2000). Further, the effect is also different to the males in Study 1; it is possible that any of the differences between participants in Study 1 and 2 (e.g., age, location, education) might contribute to this result. Further research will be required to replicate and explain this observation.

The absence of group interactions for the Recall P2 suggests that this process is intact in HD and CU, although some differences were observed in the Recognition N340 and P540. For the N340, the increase in amplitude for male HD relative to DNC (not seen in females), and particularly the abnormal increase for List A and B words (see Figure 5), suggests some difficulties with familiarity-based recognition in this group. The HD also displayed a significant increase in P540 amplitude relative to DNC, possibly suggesting greater use of recollection-based recognition in this group.

Despite the lack of behavioral effects for CU relative to HD, we nonetheless observed some differences in their ERP components. The global reduction in N340 amplitude for CU relative to HD may be due to two factors: female CU appear to show an absence of this component (compare female CU with female HD and DNC in Figure 5), while male CU appear to show a normal amplitude in comparison with the abnormal increase in HD (again, compare male DNC, HD and CU in Figure 5). Lastly, although we note that the List B vs. New comparison for P540 is confounded with familiarity, the tendency for a smaller List B > New effect for this component in CU, relative to HD (who did not differ from DNC) suggests particular cannabis-related problems in the recollection component, independent of alcohol use. Further research will be required to confirm whether this as yet non-significant result can be replicated.

## General discussion

A vast literature has investigated memory deficits using performance on the RAVLT in cannabis users and heavy drinkers. In two studies, we have extended the previous literature in reporting the first studies of event-related potentials in drug-naïve controls, let alone substance-using groups, and together with behavioral measures, examined the deficits associated with typical alcohol and/or cannabis use in young adults.

There are considerable differences between the samples collected, not only in age, but also in exposure to the drugs of interest, and location—relevant for both socioeconomic status and the recording settings in the individual laboratories, which necessitated some minor differences in early steps of ERP analysis. Despite this, we have confirmed some similarities in results between studies—notably, that while verbal memory performance in our modified RAVLT was slightly lower than published norms, the typical changes over trials remained, and demonstrably similar PCA components were extracted in each dataset. With the exception of the Recognition N340 in Study 2, these components displayed the expected topographies and condition effects. We thus have provided proof of concept that with a few modifications to the delivery of the task, the RAVLT, a widely-used, easy to administer, and normed test of learning and memory, can be extended for use in psychophysiological contexts.

With regards to substance-related effects investigated in Study 2, both the ERP and behavioral measures suggest intact immediate recall processes (Trials I-VI), but problems in HD and CU groups concerning forgetting after a delay, and for ERP but not behavioral indices of (delayed) recognition memory. For the traditional behavioral measures (learning over Trials I-V, proactive and retroactive interference), we observed only non-significant trends for group effects (sometimes interacting with sex) in Study 2; in addition we observed no differences and small effect sizes for Recall P185 amplitudes between groups. In contrast, HD displayed significantly increased forgetting after a delay, and significantly increased amplitude of the recollection-based component (P540) despite intact recognition performance. CU displayed significantly reduced amplitude of the familiarity-based N340 component overall, and a non-significant tendency for reduced amplitude of the recollection-based P540 to List B words, also despite intact recognition performance. Thus, measurement of ERPs has added value to the study of memory processes in the RAVLT, being more sensitive than performance measures to alcohol-related impairments in recognition processes (specifically, recollection), and showing that cannabis use is associated with impairments in both recollection and familiarity-based recognition processes, again despite no statistically significant deficit on behavioral measures. The lack of memory deficits in CU is peculiar, given the robust deficits demonstrated elsewhere (e.g., Yücel et al., 2008; Solowij et al., 2011); we discussed this earlier as being possibly due to the lower level of cannabis exposure in our sample. Future research should urgently investigate ERPs in the RAVLT among a sample of heavier users of cannabis.

In summary, we have demonstrated the feasibility of measuring meaningful and reliable ERP components in the RAVLT, and its sensitivity in detecting alcohol- and cannabis-related deficits not apparent in performance measures. These studies invite replication of these methods in other laboratories, and lead the way for further ERP research investigating substance-related and other memory deficits, including the effects of age of onset, level of exposure, and interactions with sex.

## Author contributions

JS and RM conceived of the study, with input from RB, AM, AF and NS. AM, RB, MD and TB collected the data for Study 1, while JS and JI collected the data for Study 2. JS and FD analyzed the ERP data and JS performed statistical analysis. All authors contributed to and approved of the final version of the manuscript.

### Conflict of interest statement

The authors declare that the research was conducted in the absence of any commercial or financial relationships that could be construed as a potential conflict of interest.
