# Supplementary material for: Verbal Learning and Memory in Cannabis and Alcohol Users: An Event-Related Potential Investigation
Source: Front Psychol. 2017 Dec 8;8:2129. doi: 10.3389/fpsyg.2017.02129 (PMC5727079; doi:10.3389/fpsyg.2017.02129)
Supplement: Supplementary file 1 [file DataSheet1.docx]

**Verbal learning and memory in cannabis and alcohol users: An event-related potential investigation**

**Supplementary Material**

Janette L. Smith, Frances M. De Blasio, Jaimi M. Iredale, Allison Matthews, Raimondo Bruno, Michelle Dwyer, Tessa Batt, Allison M. Fox, Nadia Solowij & Richard P. Mattick

There is some evidence for other ERP ‘differences based on later memory’ at the encoding stage beyond the P2 covered in the main article, but they were not the main focus of our investigation and could not be included in the main article for space reasons. Later recalled words are associated not only with a larger P2 component (e.g., Babiloni et al., 2010; Chapman et al., 1978; Smith, 1993), but also a smaller (less negative) N400 (e.g., Dunn et al., 1998; Fox et al., 1995), and a larger late positivity from about 500-900ms post-stimulus (Blanchet et al., 2007; Dunn et al., 1998; Fox et al., 1995). Cannabis-related differences in these components have been reported by our group previously (Battisti et al., 2010). Our PCAs for both adolescents and young adults identified such components in the Recall phase. We also present analyses for the additional PCA derived components identified within the P2 latency range in each analysis.

Furthermore, the adolescents recruited in Study 1 were selected to include a range of alcohol and cannabis consumption behaviours; however, since the samples were small and the alcohol and cannabis user groups were not particularly heavy users, we did not expect to observe large/significant differences between groups. However, to spur interest in future research using this paradigm in these populations, we also include group analyses for behavioural and ERP data from this study as Supplementary Material.

# Study 1: Adolescents

## Participants

The 33 participants described in the main article were recruited into three groups: ‘Heavy Drinkers’ (HD, n = 12) were defined as those who had engaged in heavy drinking (four or more Australian standard drinks, equal to 40g alcohol, on one occasion) at least once in the preceding 12 month period, but reported no previous use of cannabis. ‘Cannabis Users’ (CU, n = 8) were defined as those who reported cannabis use at least once in the last 12 months. Two of these participants reported using cannabis 2-5 times, the remaining 6 reported using 6 or more times; all CU also reported heavy drinking in the preceding 12 months. Participants who neither engaged in heavy drinking nor cannabis use were in the ‘Drug-Naive Controls’ (DNC, n = 13) group. We focused on a single sex to reduce heterogeneity in this pilot study, and on males since the prevalence of alcohol and cannabis use is slightly higher than in females in this age range (AIHW, 2011). Our aim was not to capture the heaviest users in their cohort, but rather to examine the possibility of cognitive deficits among ‘typical’ users for this age range: approximately 58.5% of males aged 16-19 have at least one alcohol binge episode in a 12 month period, while, by interpolation, around 19.5% of males in this age range have used cannabis at least once in the past year (AIHW, 2011).

## ERPs and PCA results

Supplementary Figure 1 (top) shows the grand mean waveforms in the Recall phase for Remembered and Not Remembered words, while Supplementary Figure 2 shows the unscaled factor loadings associated with each of the major components identified by the PCA. In addition to the P175 described in the main text, the PCA identified two other components in the P2 range (Factor 4, peaking at 210ms, Fz maximum, 6.49% of variance, labelled P210; and Factor 8, peaking at 260ms, P4 maximum, explaining 3.64% of variance, labelled P260). Additionally, the first factor showed a negative peak at 440ms, maximal at Fz, and explained 29.35% of variance (labelled N440), and Factor 2 showed a positive peak at 630ms, maximal at Pz and explained 15.31% of variance (labelled P630). Peak component amplitudes for these additional factors were analysed in a similar stepwise fashion as described in the main article.

## Statistical analysis

Statistical analysis for behavioural data proceeded as described in the main article, with the addition of a Group factor (DNC, HD, CU) in each MANOVA. Contrasts on the Group factor (for this and all other analyses) separately compared the performance of DNC with HD, and HD with CU. These group comparisons were selected because the CU group all reported heavy drinking; thus the DNC vs. HD comparison assesses the effect of heavy drinking, while the CU vs. HD comparison assesses the effect of cannabis use while controlling for heavy drinking (although we allow that there could be interactive effects of alcohol and cannabis, examination of these is beyond the scope of this study). For recognition accuracy, we ran Group ANOVAs for List A, List B, and New words separately, while for reaction time, we ran Type (List A, List B, New) × Group MANOVAs, with contrasts on the Type factor comparing List A performance with List B words, and with New words.

A two-step approach was taken with analyses of ERP data, to accurately describe the topography of the PCA-identified components, and to assess the important Group main effects and interactions. First, peak component amplitudes from the sites F3, Fz, F4, C3, Cz, C4, P3, Pz and P4 were each assessed with three-way MANOVAs with factors Lateral (left/midline/right), Sagittal (frontal/central/parietal) and Type (for the recall phase: Remembered, Not Remembered; for the recognition phase: List A, List B, New). Contrasts on the Sagittal factor compared activity at frontal sites with that at parietal sites, and their average with activity at central sites. Contrasts on the Lateral factor compared activity at left hemisphere sites with that at right hemisphere sites, and their average with activity at midline sites. Such contrasts are optimal for efficiently deriving maximal information about component topography. For the recognition phase, planned contrasts on the Type factor for N415 (indexing familiarity) compared activity for List A vs. List B words (highly familiar words vs. less so), and their mean (words which had been presented before) vs. New (not seen before). For the P640 (indexing recognition), we compared List A words with the mean of List B and New words (indicating correct source recollection of the word as being List A vs. Other), and compared List B with New words (although this is necessarily confounded with familiarity). The results of this step are important for characterising the topographic distribution of the component, and differences in amplitude and topography between different trial types.


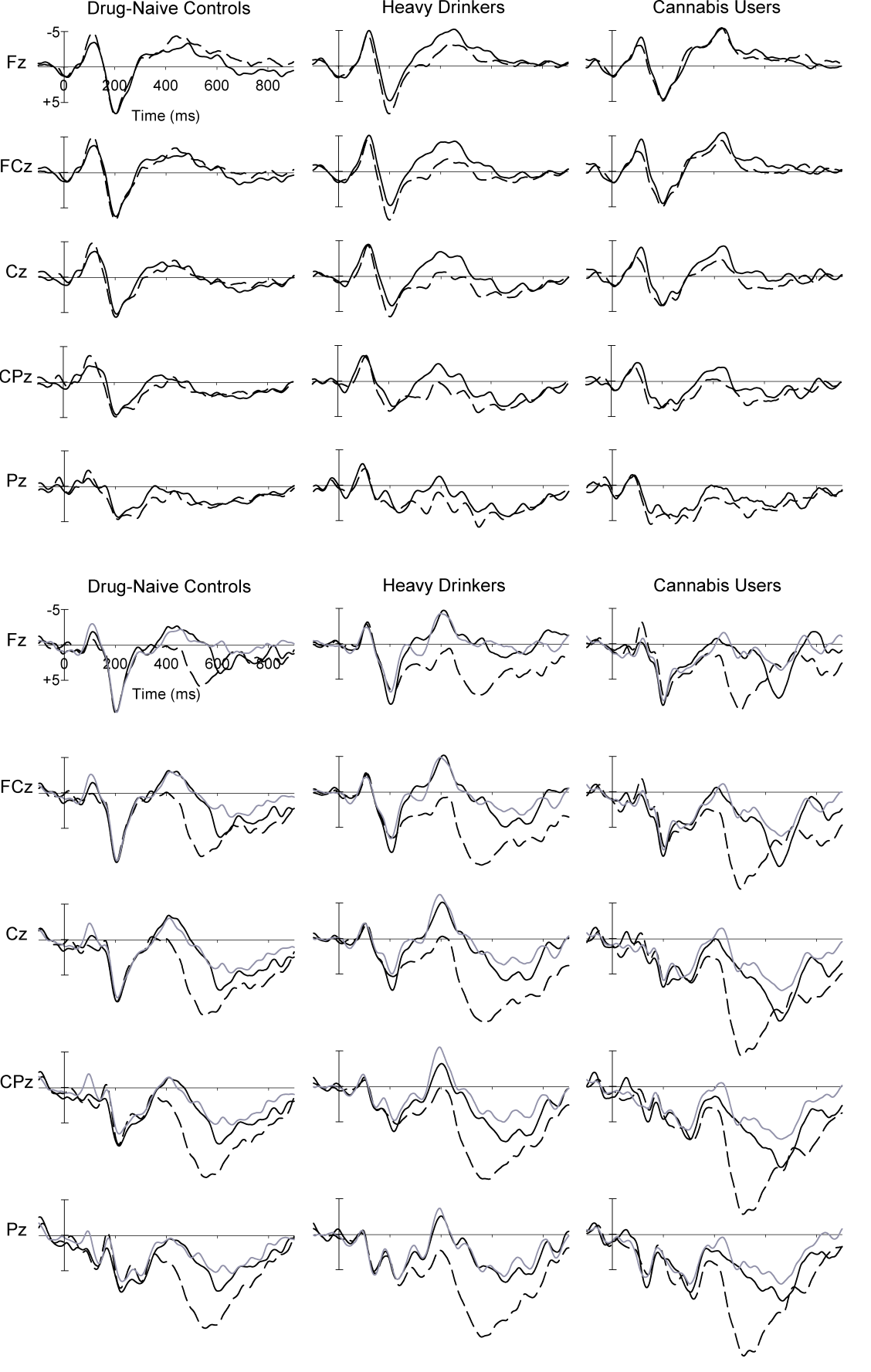


**Supplementary Figure 1**. Grand mean ERPs at midline sites for (top panel) words which were later Remembered (dashed) and Not Remembered (solid) in the Recall phase, and (bottom panel), List A (dashed), List B (black solid) and New (grey solid) words in the Recognition phase, for adolescent males (Study 1) in the Drug-Naïve Control (DNC), Heavy Drinker (HD) and Cannabis User (CU) groups.


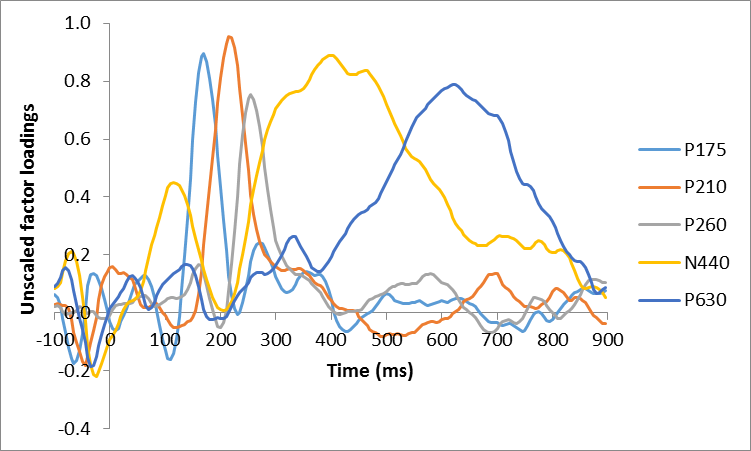


**Supplementary Figure 2**. Unscaled factor loadings over time for the Recall phase for adolescent males in Study 1.

In the second step, the average activity was calculated from a number of sites identified via the above step as the regions of maximum amplitude, and these single variables (one for each component) were entered into separate Type × Group MANOVAs. Contrasts on the Group and Type factors were as mentioned above. As the contrasts were planned and there were no more of them than the degrees of freedom for effect, no Bonferroni-type adjustment to alpha was necessary (Tabachnick and Fidell, 2001). Because this is a first step in examining ERPs in the RAVLT, with a small sample size and low power, but with an aim to report potential discoveries to spur future research, we report any effect with *p<*0.100.

## Demographics

Supplementary Table 1 displays the demographic information for each group in the study. The groups were well matched for age (*p>*0.643), and for the proportion of right handed participants (*χ^2^*(2)=1.19, *p=*0.552). DNC and HD were mostly non-smokers, with one participant in each group reporting smoking tobacco on 2-5 occasions (the remainder reporting never smoking) in the previous 12 months, while 6 CU reported smoking on 6 or more occasions in the previous 12 months (the other two reporting smoking once or never; *χ^2^*(2)=27.79, *p<*0.001). One participant in the CU group reported one-time use of ecstasy, another reported one-time use of speed, and a third reported one-time use of LSD. Standardised scores on the WTAR were in the normal range and not significantly different between groups (both *p>*0.494). Thus, we recruited a relatively homogenous sample of participants, who differ on alcohol and cannabis use according to the selection criteria, and who represent ‘typical’ users of their age (AIHW, 2011), rather than heavy users.

**Supplementary Table 1**. Demographic information and behavioural performance means for Drug-Naïve Controls (DNC), Heavy Drinkers (HD) and Cannabis Users (CU) in the sample of adolescent males (Study 1).

|  | DNC  (n = 13) | | HD  (n = 12) | | CU  (n = 8) | |
| --- | --- | --- | --- | --- | --- | --- |
|  | Mean | SD | Mean | SD | Mean | SD |
| Age (years) | 17.3 | 0.7 | 17.2 | 0.7 | 17.1 | 0.8 |
| % Right handed | 83.3 |  | 92.3 |  | 75.0 |  |
| WTAR standardised score | 101.1 | 16.7 | 101.0 | 15.1 | 107.0 | 19.5 |
| **Recall phase performance** |  |  |  |  |  |  |
| Trial I | 4.8 | 1.6 | 5.4 | 1.0 | 5.9 | 1.6 |
| Trial II | 7.8 | 1.9 | 7.9 | 1.9 | 7.9 | 3.3 |
| Trial III | 8.7 | 2.6 | 10.0 | 1.8 | 10.0 | 2.9 |
| Trial IV | 10.0 | 3.1 | 11.3 | 1.9 | 10.9 | 1.7 |
| Trial V | 11.1 | 1.9 | 11.3 | 2.3 | 11.8 | 2.4 |
| Trial B | 4.4 | 1.6 | 4.4 | 1.6 | 4.8 | 1.4 |
| Trial VI | 9.7 | 3.1 | 10.0 | 2.4 | 11.5 | 1.9 |
| Trial VII | 9.6 | 2.7 | 9.3 | 2.2 | 10.3 | 2.6 |
| Total words recalled (Trials I-V) | 42.4 | 9.3 | 45.9 | 6.4 | 46.4 | 10.8 |
| Learning rate (V minus I) | 6.3 | 1.9 | 5.8 | 2.6 | 5.9 | 2.2 |
| Proactive interference (I minus B) | 0.4 | 2.3 | 1.0 | 1.7 | 1.1 | 2.3 |
| Retroactive interference (V minus VI) | 1.4 | 2.0 | 1.3 | 1.9 | 0.3 | 1.9 |
| Forgetting (V minus VII) | 1.5 | 2.1 | 1.9 | 1.5 | 1.1 | 2.5 |
| **Recognition phase performance** |  |  |  |  |  |  |
| List A accuracy (number correct/15) | 13.6 | 1.5 | 14.2 | 0.9 | 12.3 | 4.0 |
| List B accuracy (number correct/15) | 14.3 | 0.9 | 13.8 | 1.4 | 13.8 | 1.7 |
| New accuracy (number correct/20) | 18.4 | 1.4 | 18.8 | 2.1 | 17.5 | 2.3 |
| List A RT (ms) | 903.7 | 208.6 | 967.5 | 444.7 | 733.5 | 117.3 |
| List B RT (ms) | 894.2 | 193.8 | 951.3 | 218.4 | 749.7 | 155.6 |
| New RT (ms) | 933.4 | 149.3 | 1003.8 | 295.1 | 823.8 | 218.4 |

* N = 7 for the CU group, since one participant performed poorly on the recognition task such that reliable ERPs could not be calculated.

## Behavioural performance

The within-subject effects described in the main article were upheld: that is, typical effects of learning over trials, proactive and retroactive interference, and forgetting after a delay, were all observed. However, there were no significant effects or interactions involving group (*df* = 1,31) for learning over trials (all *p*>0.321), proactive interference (all *p*>0.417), retroactive interference (all *p*>0.268) and forgetting (all *p*>0.365). Similarly, small-medium effect sizes were observed for all comparisons (total words recalled, DNC vs. HD: Cohen’s *d*=0.440; HD vs. CU: *d*=0.055; proactive interference, DNC vs. HD: *d*=-0.300; HD vs. CU: *d*=0.064; retroactive interference DNC vs. HD: *d*=0.069; HD vs. CU: *d*=0.531; forgetting, DNC vs. HD, *d*=-0.250, HD vs. CU: *d*=0.408; in all cases, a negative effect size represents poorer performance in the HD than DNC group, or in the CU than HD group). There were also no differences between groups in the recognition accuracy rates for List B (all *p*>0.282) or New words (all *p*>0.162), but CU correctly categorised marginally fewer List A words compared to HD (*F*=3.62, *p*=0.067, *d*=-0.745). Responses in the recognition phase were faster for CU than HD (*F=*4.18, *p=*0.050, *d*=0.829) across stimulus types, but there were no other significant effects.

## Recall ERP components

The results of the topographic analyses of the P175 component are reported in the main text. For the second ANOVA, the average of sites F3, Fz, F4, FC3, FCz and FC4 was entered into the Type × Group ANOVA. The Type main effect remained significant (*F=*8.07, *p=*0.008; all *df=*1,30, see Supplementary Figure 3 for topographic maps of activity), but there were no significant effects of group (magnitude of Remembered > Not Remembered effect difference between groups DNC vs. HD: *F*=0.37, *p*=0.550, *d*=0.242; HD vs. CU: *F*=0.62, *p*=0.435, *d*=0.366; see Table 1 for mean and SD for each condition and group).

**

**

**Supplementary Figure 3.** Topographic plots of activity across sites, groups and conditions for P175, P210, P260, N440 and P630 components in the Recall phase for adolescent males (Study 1).

For the P210, a Type × Lateral × Sagittal interaction was marginally significant (*F=*3.37, *p=*0.076;), such that Not Remembered words were associated with a small midline < hemispheres effect at central sites, with equal amplitude at frontal/parietal sites, and Remembered words showed equal amplitude at central sites, with a small midline < hemispheres effect at frontal/parietal sites. No other effects approached significance. The average of sites F3, Fz, F4, FC3, FCz and FC4 were entered into the second MANOVA. The only effect approaching significance was a Group × Type interaction (*F=*3.27, *p=*0.081), such that HD and DNC showed similar amplitude for Remembered words, but HD showed a reduction for Not Remembered words while DNC showed an increase.

The P260 was marginally larger at parietal than frontal sites (*F=*3.24, *p=*0.081), and was larger in the hemispheres compared to the midline (*F=*26.42, *p<*0.001). The average of sites CP3, CP4, P3 and P4 was entered into the second MANOVA. A Group × Type interaction was significant (*F=*4.25, *p=*0.048), such that DNC and HD showed similar amplitudes to Not Remembered words, while for Remembered words, DNC showed a slight increase in amplitude, while HD showed a marked decrease. No other interactions were significant.

The N440 was more negative at frontal than parietal sites (*F=*66.95, *p<*0.001), and more negative centrally than at frontal/parietal sites (*F=*19.89, *p<*0.001; see Supplementary Figure 2 for plots of topographic activity across trial types and groups for the N440 and P630). Amplitudes were more negative in the midline than hemispheres (*F=*41.26, *p<*0.001). Increased negativity on the right compared to left was greater at parietal than frontal sites (*F=*4.53, *p=*0.041), and the midline > hemispheres effect was somewhat greater at parietal than frontal sites (*F=*3.31, *p=*0.078), and much larger at central than frontal/parietal sites (*F=*4.90, *p=*0.034). The N440 was more negative for Not Remembered than Remembered words (*F=*8.89, *p=*0.005). The frontal > parietal gradient was marginally greater for Remembered than Not Remembered words (*F=*3.57, *p=*0.068), while the central > frontal/parietal effect was greater for Not Remembered than Remembered words (*F=*4.43, *p=*0.043). For Not Remembered words, the midline > hemispheres effect was greater at parietal than frontal sites, while for Remembered words, the effect was of similar amplitude parietally and frontally (*F=*5.93, *p=*0.021). The average of sites F3, Fz, F4, FC3, FCz and FC4 was entered into the second MANOVA. The Type main effect was marginally significant (*F=*2.99, *p=*0.094), and Group × Type interactions were significant: the HD group showed a much larger N440 to Not Remembered than Remembered words, in contrast to DNC (*F=*4.38, *p=*0.045) and CU (*F=*3.98, *p=*0.055), which both showed similar amplitudes to Remembered and Not Remembered words.

The P630 was more positive parietally than frontally (*F=*38.67, *p<*0.001), and at central compared to frontal/parietal sites (*F=*52.17, *p<*0.001). P630 was larger at right than left hemisphere sites (*F=*5.50, *p=*0.025), and larger still in the midline (*F=*21.77, *p<*0.001). The right > left effect was marginally greater at parietal than frontal sites (*F=*3.37, *p=*0.076). The midline > hemispheres effect was greater at parietal than frontal sites (*F=*37.74, *p<*0.001), and at central compared to frontal/parietal sites (*F=*4.81, *p=*0.036). The central > frontal/parietal effect was greater for Remembered than Not Remembered words (*F=*6.37, *p=*0.017), as was the parietal > frontal × right > left effect (*F=*3.55, *p=*0.069). The average of sites Cz, CPz, CP4 and Pz was entered into the second MANOVA. Only a group main effect was of marginal significance: P630 tended to be larger in HD than DNC overall (*F=*2.92, *p=*0.098).

## Recognition ERP components

For both the Recognition N415 and Recognition P640 components, topographic results are presented in the main text; topographic maps for each group are displayed in Supplementary Figure 4. For N415, the average of sites Cz and C4 was entered into the second MANOVA. The N415 was more negative for New than List A/B words (*F=*5.35, *p=*0.028; *df=*1, 29 for the Group × Type analyses for this and P640), and more negative for List B than List A words (*F=*15.49, *p<*0.001), but there were no significant effects of group.





**Supplementary Figure 4.** Topographic plots of activity across sites, groups and conditions for the N415 and P640 components in the Recall phase for adolescent males (Study 1).

For the P640, the average of sites Cz, CPz, P3 and Pz was entered into the second MANOVA. List A remained greater in positivity than Other words (*F=*11.84, *p=*0.002), and List B showed greater positivity than New words (*F=*6.62, *p=*0.015), but there were no significant group effects.

# Study 2: Young adults

## PCA results

Supplementary Figure 5 shows the unscaled factor loadings associated with each of the major components identified by the Recall PCA. In addition to the P185 described in the main text, the PCA extracted another component in the P2 range (Factor 4, labelled P255: P6 maximum, explaining 8.49% of variance), as well as a negative component peaking at 380ms (Factor 2, labelled N380, maximal at F2, explained 21.08% of variance), and a later positive component peaking at 535 ms (Factor 1, labelled P535, maximal at Pz, explained 23.02% of variance). Topographic maps of activity can be seen in Supplementary Figure 6. Peak data from these components were entered into similar two-step analyses as described in the main text.


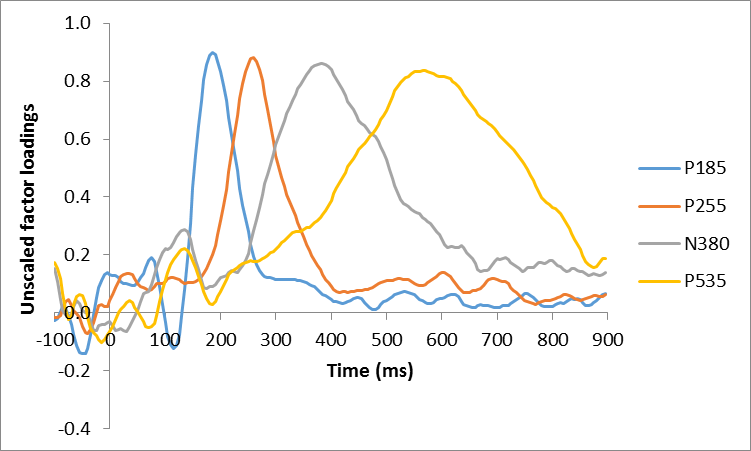


**Supplementary Figure 5**. Unscaled factor loadings over time for the Recall phase for female and male young adults in Study 2.

## Recall ERP components

The P255 showed a right > left effect (*F=*5.99, *p=*0.016) and a hemispheres > midline effect (*F=*29.30, *p<*0.001), which was stronger at parietal than frontal sites (*F=*47.87, *p<*0.001). The average of CP3, CP4, P3 and P4 were entered into the second MANOVA, which showed no significant effects.

N380 showed greater negativity at frontal than parietal sites (*F=*81.89, *p<*0.001), and at central compared to frontal/parietal sites (*F=*29.19, *p<*0.001). Amplitudes were more negative in the midline than hemispheres (*F=*105.52, *p<*0.001). The midline > hemispheres effect was greater at parietal compared to frontal sites (*F=*29.29, *p<*0.001) and at central compared to frontal/parietal sites (*F=*32.16, *p<*0.001). N380 was more negative for Not Remembered than Remembered words (*F=*69.23, *p<*0.001), and the frontal > parietal gradient was larger for Remembered words (*F=*20.86, *p<*0.001), while the central > frontal/parietal effect was larger for Not Remembered words (*F=*5.87, *p=*0.017). Further, the midline > hemispheres effect was larger for Not Remembered than Remembered words (*F=*26.78, *p<*0.001). The parietal > frontal × midline > hemispheres effect was greater for Not Remembered than Remembered words (*F=*18.19, *p<*0.001). The average activity across sites F1, Fz, F2, FC1, FCz, and FC2 were entered into the second MANOVA. N380 was greater for Not Remembered than Remembered words (*F=*34.93, *p<*0.001), especially in females compared to males (*F=*4.03, *p=*0.047). N380 was marginally smaller in the CU compared to HD (*F=*3.32, *p=*0.072, consistent with Battisti et al., 2010), who did not differ from DNC (*F=*2.28, *p=*0.134).

P535 showed a parietal > frontal effect (*F=*171.88, *p<*0.001), and a central > frontal/parietal effect (*F=*61.41, *p<*0.001). The component was more positive in the midline than hemispheres (*F=*35.01, *p<*0.001). Parietally, a right > left effect was observed, while a

**

**

**Supplementary Figure 6.** Topographic plots of activity across sites, groups and conditions for P255, N380 and P535 components in the Recall phase for female and male young adults (Study 2).

reversed and reduced effect was observed at frontal sites (*F=*5.28, *p=*0.024); further, the midline > hemispheres effect was greater parietally than frontally (*F=*15.92, *p<*0.001). The main effect of Type was not significant (*F=*0.00, *p=*0.971), but the parietal > frontal gradient was larger for Remembered than Not Remembered words (*F=*9.46, *p=*0.003). The average activity across sites CPz, P1, Pz, P2, and POz were entered into the second MANOVA. The main effect of Type remained non-significant (*F=*3.01, *p=*0.086), and a main effect of sex indicated larger P535 in females than males (*F=*13.04, *p<*0.001). The increase in P535 for females compared to males was larger in the CU than HD group (*F=*4.98, *p=*0.028), and a three-way interaction approached significance, such that male HD and female HD showed similar Remembered > Not Remembered effects, while amongst CU, males showed a Remembered > Not Remembered effect while females displayed increased amplitude for Not Remembered words (*F=*3.69, *p=*0.058).

## Comparison of component topographies across analyses

We desired to test the similarity of components identified across samples, beyond subjective comparisons of the component’s site of maximum amplitude, variance explained in the PCA, latency, polarity and topography. However, due to the slightly earlier peak latency in young adults compared to adolescents (a finding reported elsewhere, e.g., Tomé et al., 2015; van Dinteren et al., 2014), the data were not suited to make use of the congruence coefficient (Lorenzo-Seva and ten Berge, 2006). Instead, we calculated the average amplitude across groups and conditions (Remembered and Not Remembered for the Recall phase, and List A, List B and New words for the Recognition phase) at each of the 30 recorded sites which were common to both datasets, and compared the topographies between the adolescent and young adult datasets using Pearson correlations (Barry et al., 2016). Correlations were universally positive and of large magnitude (all *p<*0.001), indicating high concordance between topographies in each sample: adolescent recall P175 with young adult recall P185 r(28) = 0.91; adolescent recognition N415 with young adult recognition N340 r(28)=0.89; adolescent recognition P640 with young adult recognition P540 r(28)=0.86). The similarity in topography suggests that brain generators underlying these components change little with age from adolescence to young adulthood.

**References**

Australian Institute of Health and Welfare (AIHW) 2011. 2010 National Drug Strategy Household Survey report. Australian Institute of Health and Welfare, Canberra.

Babiloni, C., Vecchio, F., Mirabella, G., Sebastiano, F., Gennaro, G.D., Quarato, P.P., Buffo, P., Esposito, V., Manfredi, M., Cantore, G., Eusebi, F., 2010. Activity of hippocampal, amygdala, and neocortex during the Rey auditory verbal learning test: An event-related potential study in epileptic patients. Clin Neurophysiol 121, 1351-1357.

Barry, R.J., De Blasio, F.M., Cave, A.E., 2016. Sequential processing in young and older adults in the equiprobable auditory Go/NoGo task. Clin Neurophysiol 127, 2273-2285.

Battisti, R.A., Roodenrys, S.J., Johnstone, S.J., Respondek, C., Hermens, D.F., Solowij, N., 2010. Chronic use of cannabis and poor neural efficiency in verbal memory ability. Psychopharmacol 209, 319-330.

Blanchet, S., Gagnon, G., Bastien, C., 2007. Event-related potential study of dynamic neural mechanisms of semantic organizational strategies in verbal learning. Brain Res 1170, 59-70.

Chapman, R.M., McCrary, J.W., Chapman, J.A., 1978. Short-Term Memory: The "Storage" Component of Human Brain Responses Predicts Recall. Science 202, 1211-1214.

Dunn, B.R., Dunn, D.A., Languis, M., Andrews, D., 1998. The Relation of ERP Components to Complex Memory Processing. Brain Cogn 36, 355-376.

Fox, A.M., Michie, P.T., Coltheart, M., Solowij, N., 1995. Memory functioning in social drinkers: A study of Event-Related Potentials. Alcohol Alcohol 30, 303-310.

Lorenzo-Seva, U., ten Berge, J.M.F., 2006. Tucker's Congruence Coefficient as a meaningful index of factor similarity. Methodol 2, 57-64.

Smith, M.E., 1993. Neurophysiological Manifestations of Recollective Experience during Recognition Memory Judgments. J Cogn Neurosci 5, 1-13.

Tomé, D., Barbosa, F., Nowak, K., Marques-Teixeira, J., 2015. The development of the N1 and N2 components in auditory oddball paradigms: a systematic review with narrative analysis and suggested normative values. J Neural Transm 122, 375-391.

van Dinteren, R., Arns, M., Jongsma, M.L.A., Kessels, R.P.C., 2014. P300 Development across the Lifespan: A Systematic Review and Meta-Analysis. PLoS ONE 9, e87347.
